# GABA, Glutamate and Neural Activity: A Systematic Review With Meta-Analysis of Multimodal ^1^H-MRS-fMRI Studies

**DOI:** 10.3389/fpsyt.2021.644315

**Published:** 2021-03-08

**Authors:** Amanda Kiemes, Cathy Davies, Matthew J. Kempton, Paulina B. Lukow, Carly Bennallick, James M. Stone, Gemma Modinos

**Affiliations:** ^1^Psychosis Studies Department, Institute of Psychiatry, Psychology and Neuroscience, King's College London, London, United Kingdom; ^2^Department of Neuroimaging, Institute of Psychiatry, Psychology and Neuroscience, King's College London, London, United Kingdom; ^3^Brighton and Sussex Medical School, University of Sussex & University of Brighton, Brighton, United Kingdom; ^4^Medical Research Centre Centre for Neurodevelopmental Disorders, King's College London, London, United Kingdom

**Keywords:** glutamate, GABA, magnetic resonance spectroscopy, fMRI, multimodal neuroimaging

## Abstract

Multimodal neuroimaging studies combining proton magnetic resonance spectroscopy (^1^H-MRS) to quantify GABA and/or glutamate concentrations and functional magnetic resonance imaging (fMRI) to measure brain activity non-invasively have advanced understanding of how neurochemistry and neurophysiology may be related at a macroscopic level. The present study aimed to perform a systematic review and meta-analysis of available studies examining the relationship between ^1^H-MRS glutamate and/or GABA levels and task-related fMRI signal in the healthy brain. Ovid (Medline, Embase, and PsycINFO) and Pubmed databases were systematically searched to identify articles published until December 2019. The primary outcome of interest was the association between resting levels of glutamate or GABA and task-related fMRI. Fifty-five papers were identified for inclusion in the systematic review. A further 22 studies were entered into four separate meta-analyses. These meta-analyses found evidence of significant negative associations between local GABA levels and (a) fMRI activation to visual tasks in the occipital lobe, and (b) activation to emotion processing in the medial prefrontal cortex (mPFC)/anterior cingulate cortex (ACC). However, there was no significant association between mPFC/ACC glutamate levels and fMRI activation to cognitive control tasks or to emotional processing, with the relationship to emotion processing related neural activity narrowly missing significance. Moreover, our systematic review also found converging evidence of negative associations between GABA levels and local brain activity, and positive associations between glutamate levels and distal brain activity, outside of the ^1^H-MRS sampling region. Albeit less consistently, additional relationships between GABA levels and distal brain activity and between glutamate levels and local brain activity were found. It remains unclear if the absence of effects for other brain regions and other cognitive-emotional domains reflects study heterogeneity or potential confounding effects of age, sex, or other unknown factors. Advances in ^1^H-MRS methodology as well as in the integration of ^1^H-MRS readouts with other imaging modalities for indexing neural activity hold great potential to reveal key aspects of the pathophysiology of mental health disorders involving aberrant interactions between neurochemistry and neurophysiology such as schizophrenia.

## Introduction

Excitation-inhibition balance plays a major role in determining neural activity (1). At a microscopic level, the influence of the brain's major excitatory and inhibitory neurotransmitters [glutamate and γ-aminobutyric acid (GABA)], on neural activity has been studied in detail [see Isaacson and Scanziani (1), Lauritzen et al. (2)]. At a macroscopic level, this investigation has been achieved through the development and optimisation of neuroimaging techniques enabling quantification of GABA and glutamate concentrations via proton magnetic resonance spectroscopy (^1^H-MRS), and of neural activation via measurement of blood oxygen level dependent (BOLD) signal with functional magnetic resonance imaging (fMRI). BOLD signal has been linked to local field potentials of dendritic origins, implying the signal is likely to reflect incoming input (3), thereby being closely linked to, and affected by, neurotransmission. However, the relationship between neurotransmitter levels and task-related neural activity remains unclear.

A previous narrative review by Duncan et al. (4) sought to address this question with a comprehensive overview of studies using imaging techniques such as magnetoencephalography, electroencephalography, fMRI, positron emission tomography and ^1^H-MRS, as well as behavioural measures as proxies for neural activity and pharmaceutical manipulations of neurotransmitter levels. The authors reported that GABA levels were related to reduced positive BOLD response or reduced negative BOLD response (NBR) within the same brain region, while glutamate levels were more commonly related to inter-regional neural responses. The number of publications using multimodal fMRI and ^1^H-MRS techniques has more than doubled since this review was published, and studies using this type of multimodal combination are expected to keep increasing in the future due to the ease of collecting both types of data within a single scanning session. More recent reports outside of the previous review include studies on regions of interest that had previously been examined too infrequently to permit meta-analysis, providing important insights into the nature of neurochemistry-neurophysiology associations in a variety of brain regions and task domains.

The present study aims to address these issues by conducting a systematic review and meta-analysis of ^1^H-MRS and task-based fMRI studies in the healthy brain. We then meta-analyse homogeneous studies based on the location of ^1^H-MRS voxel placement and fMRI paradigm. Finally, we discuss the findings in light of abnormalities in these relationships in psychiatric populations, such as schizophrenia patients, and their potential implications on brain processes in psychiatric disorders.

## Methods

Systematic searches were performed on Pubmed and Ovid (Medline, Embase and PsycINFO) databases from database inception to 4 December 2019. Search strategies for the individual databases included the terms “functional magnetic resonance imaging” AND “magnetic resonance spectroscopy” AND (“GABA” OR “glutamate”) (for full search syntax see Supplementary Table 1). We included studies that used ^1^H-MRS to measure GABA, glutamate or Glx (combined glutamate and glutamine) metabolite levels to test associations with task-related fMRI activation. Eligible study designs included observational studies, cohort studies, case-control studies, cross-sectional studies, or experimental studies (for full eligibility criteria see Supplementary Table 2). The population of interest were healthy human participants, or healthy human control groups from case-control studies. Interventional studies were excluded, unless those studies conducted and reported the outcome of interest analysis with baseline measures. Studies assessing only changes in metabolite levels before, during and after a task together with BOLD activation were not included as they do not investigate the relationship between resting-state neurotransmitter levels and neural activity modulated by stimuli. Two reviewers (AK and CB) independently screened search result titles and abstracts of papers for eligibility according to the inclusion and exclusion criteria. All potentially eligible studies were screened full-text for inclusion. Reference lists of eligible studies were hand-searched for further relevant articles.

Two independent reviewers (AK and PBL) extracted data using a standardised template datasheet. The primary outcomes extracted were the metabolite type, ^1^H-MRS metabolite sampling region, fMRI task paradigm, neural activation region, and relationship between metabolite and neural activity. Studies investigating the relationship between ^1^H-MRS GABA or glutamate and task-related BOLD signal performed analyses in one or both of two different ways: by including metabolite levels as a covariate in a regression, or by extracting the percentage of BOLD signal change (%SC) from a region of interest (ROI) and using correlation analysis with the metabolite levels. Both types of analysis methods were included in this review. Regressions either covered the whole-brain or a predetermined ROI such as the ^1^H-MRS voxel, an anatomical ROI or a mask derived from an activation cluster. Correlation analyses used %SC extracted from sources such as the peak voxel within or mean activation across an activated cluster, an anatomically defined ROI, or the ^1^H-MRS voxel. Additional extracted data for the systematic review included the statistical significance of the relationship, covariates tested in the analysis, sample size, age and sex characteristics of the sample, scanner strength, ^1^H-MRS sequence, and reference metabolite. In cases where no statistical significance value was reported for the relationship itself and only Pearson's *r* correlation coefficients were reported [e.g., (5)], *p-*values were calculated through conversion of r values to test statistic *t* with the formula

$$\begin{array}{r} t=\frac{r}{\sqrt{\left(\left(1-r^{2}\right)/\left(df\right)\right)}} \end{array}$$

The *p*-value was then derived using the sampling distribution of Student's *t*. This calculation of the *p*-value was used to ascertain the significance of the correlation coefficient.

**Figure 1:**
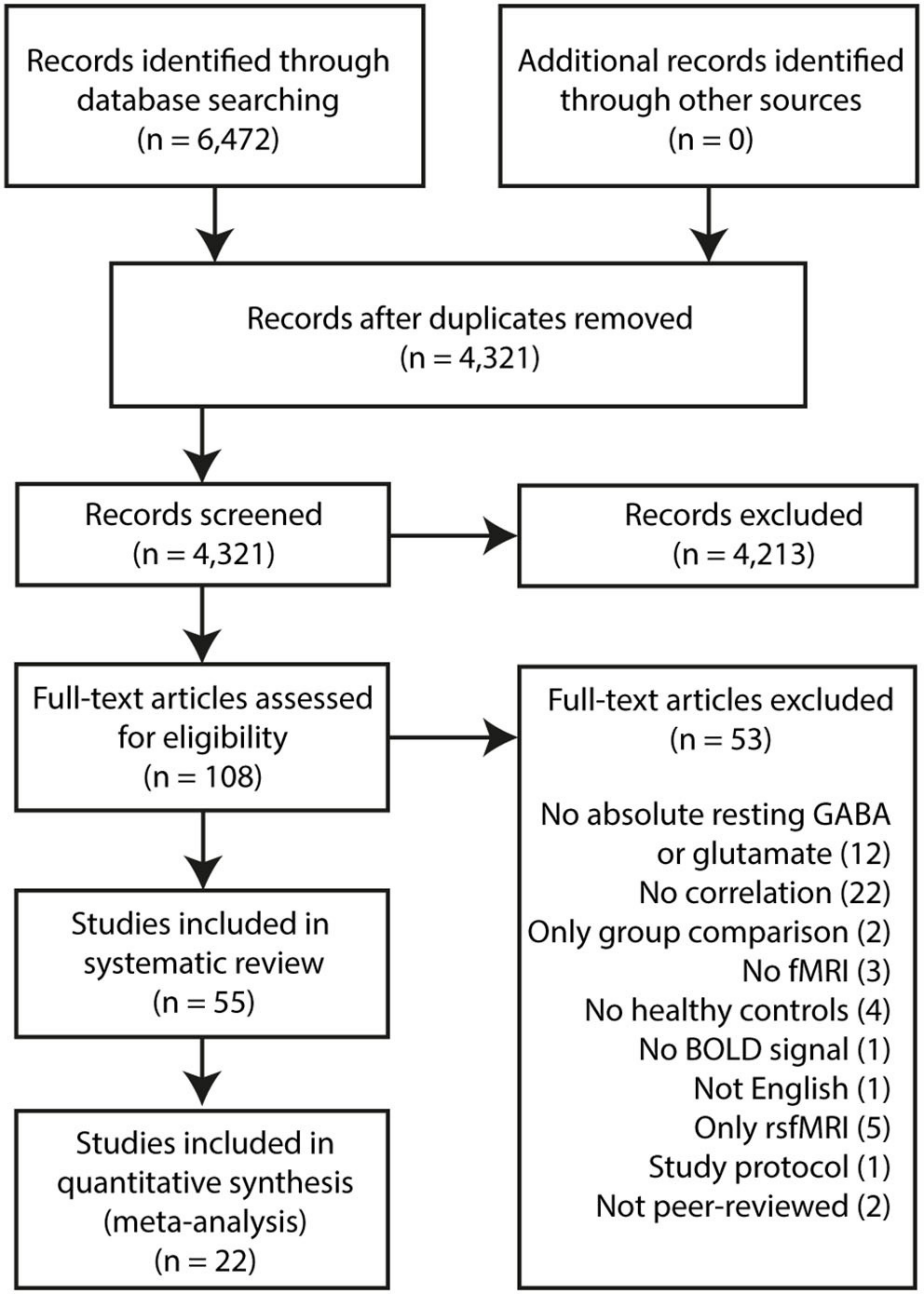
PRISMA Flowchart.

Some studies verified their main findings (^1^H-MRS x fMRI response) in the following ways: (1) substituting the metabolite levels from their primary region of interest with those from a different, control sampling region; (2) substituting the fMRI response from their primary task paradigm with that from a control task; (3) substituting the fMRI response from their primary region of interest with that from a control region during the same task. Such control analyses may be found in Supplementary Tables 7, 8. A formal quality assessment was not conducted due to a lack of standard quality assessment for such types of studies. Imaging and study design parameters were, however, collected (Supplementary Tables 3–6, 9). Review findings [see Figure 1 for PRISMA flowchart (6)] are presented according to the brain region from which metabolite levels were sampled.

### Meta-Analysis

Studies were further subdivided by ^1^H-MRS voxel location, task domain and fMRI activation foci. For quantitative analysis, *r* correlation coefficients, *t*-values, *z*-values and sample sizes were compiled. Although the recommended minimum number of studies for meta-analysis is two, this does not apply to random-effect models (7), which the MetaNSUE method employs (8). Additionally, given the likelihood of a non-statistically significant unreported effect, the minimum number of comparable studies was set at a more conservative number of ≥4. Meta-analysis was conducted using the MetaNSUE package for R (8), where r correlation coefficients were converted into effect sizes for a meta-analytic pooled effect size. This tool was used due to its ability to impute results for unreported correlation coefficients, which can often be the case when correlations are non-significant. Authors were contacted for unreported *r* correlation coefficients. However, in the case of unreported *r* correlation coefficients, the number of imputations was kept at the package's default of 500 imputations per study.

In instances where studies reported both *t*- and *z*-values of significantly correlated clusters from a regression analysis, *t*- and *z*-values were converted into *r* correlation coefficients as follows:

1. *Z*-score to *p*-value calculation: $P\left(Z\leq z\right)=\int_{-\infty }^{z}\frac{1}{\sqrt{2\pi }}e^{\frac{-\;u^{2}}{2}}du$
2. *P*-value to student *t*-value: using Microsoft Excel's TINV function
3. *T*-value to *r* correlation coefficient conversion: $r\;=\;\sqrt{t^{2}/\left(t^{2}+df\right)}$

Significance was determined using two-tailed 95% confidence intervals. Further sensitivity analyses are detailed in the Supplementary Material.

## Results

### Systematic Review of Associations With GABA and Glutamate Concentrations

#### Occipital Cortex

^1^H-MRS studies sampling GABA levels from the occipital lobe have been largely homogeneous (Table 1): using visual fMRI tasks and focusing only on co-localised neural activity. The first few studies in this domain found negative associations between occipital lobe GABA levels and local BOLD response to visual stimuli (9–12, 14). Muthukumaraswamy et al. (11) not only found a negative association to BOLD response amplitude, but also a positive correlation with BOLD response width. However, this relationship to response amplitude was not replicated in subsequent studies (13, 15, 16, 18). Harris et al. (15) investigated this relationship empirically, and combined their result with prior studies in a meta-analysis, where they found no evidence for a significant association between the two measures. More recently, Duncan et al. (16) attempted to address this issue using improved methodology. A MEGA-PRESS sequence that was adapted to suppress macromolecule signal was used so that both GABA+ and GABA– were measured from the occipital lobe. Although their empirical study found no significant relationship, Duncan et al. (16) added their results to the five studies meta-analysed in Harris et al. (15)—using the same Hunter-Schmidt method employed by the *metafor* R package—and found evidence in favour of a negative relationship between GABA and BOLD, regardless of macromolecule suppression.

**Table 1:** Occipital lobe ^1^H-MRS studies.

**Authors**	***n*** **Male %** **Age M(SD)**	**System** **Sequence**	**Reference**	**fMRI task/stimuli**	**Relationship**	**Covariates**
**GABA**						
*Medial occipital cortex*/*Visual cortex*						
Muthukumaraswamy et al. (9)	12 100 34.8	3T MEGA-PRESS	/Wt	Stationary visual grating	Visual stimuli (–) GABA	–
Donahue et al. (10)	12 50 30 (4)	3T MEGA-PRESS	/Cr	Flashing checkerboard	Visual stimuli (–) GABA+	–
Muthukumaraswamy et al. (11)	14 100 24.2 (2.4)	3T MEGA-PRESS	/Wt	Stationary visual grating	Visual stimuli (–) GABA	–
Violante et al. (12)	26 38.5 13.1 (2.9)	3T MEGA-PRESS	/Cr	Stationary visual grating	Visual stimuli (–) GABA	–
Bridge et al. (13)	13 0 30 (6)	3T SPECIAL	/tCr	Visual stimuli (diffuse light/flickering checkerboard)	Visual stimuli (#) GABA	–
Bednarík et al. (14)	12 46.7 33 (13)	7T Semi-LASER	/Wt	Flickering checkerboard	Visual stimuli (–) GABA	–
Harris et al. (15)	18 38.9 27.8 (4.0)	3T MEGA-PRESS	/Wt, /Cr	Stationary visual grating	Visual stimuli (#) GABA+	–
Duncan et al. (16)	31 0.65 23.0 (3.1)	3T MEGA-PRESS	/Wt	Stationary visual grating	Visual stimuli (#) GABA-	–
Wijtenburg et al. (17)	18 50 36.2 (16)	3T MEGA-PRESS	/Wt	Visual plasticity task	Visual plasticity (+) GABA- Baseline visual stimuli (–) GABA-	age
Costigan et al. (18)	40 30 22.1 (2.1)	3T MEGA-PRESS	/Wt	Odd-one-out task	Scene viewing (#) GABA	–
Schallmo et al. (19)	22 12 24 (3.6)	3T MEGA-PRESS	/Wt	Visual spatial suppression task	Visual spatial suppression (#) GABA+	–
*Lateral occipital cortex*/*visual motion complex*						
Schallmo et al. (19)	22 12 24 (3.6)	3T MEGA-PRESS	/Wt	Visual spatial suppression task	Visual spatial suppression (#) GABA+	–
**Glutamate**						
*Medial occipital lobe*/*Visual cortex*						
Muthukumaraswamy et al. (9)	12 100 34.8	3T MEGA-PRESS	/Wt	Stationary visual grating	Visual stimuli (#) Glx	–
Violante et al. (12)	26 38.5 13.1 (2.9)	3T MEGA-PRESS	/Cr	Moving visual grating	Visual stimuli (#) Glx	–
Bridge et al. (13)	13 0 30 (6)	3T SPECIAL	/tCr	Visual stimuli (light/flickering checkerboard)	Visual stimuli (#) Glu	–
Bednarík et al. (14)	12 46.7 33 (13)	7T Semi-LASER	/Wt	Flickering checkerboard	Visual stimuli (#) Glu	–
Ip et al. (20)	13 50 28.7 (5.6)	7T Semi-Laser	/Wt	Flickering checkerboard	Visual stimuli (#) Glu	–
Wijtenburg et al. (17)	18 50 36.2 (16)	PR-STEAM		Visual plasticity task	Visual plasticity (+) Glu	Age
Schallmo et al. (21)	22 40.9 24 (3.7)	MEGA-PRESS	/Wt	Visual spatial suppression task	Visual spatial suppression (#) Glx	–
*Lateral occipital cortex*/*visual motion complex*						
Schallmo et al. (21)	22 40.9 24 (3.7)	MEGA-PRESS	/Wt	Visual spatial suppression task	Visual spatial suppression (+) Glx	–

*Wt, Water; Cr, Creatine; (+) positive association; (#) no association; (–) negative association; GABA+, GABA+macromolecules; Glx, Glutamate + Glutamine*.

Neural activity in the occipital lobe was also studied in different contexts beyond simple visual perception. Wijtenburg (17) found a positive correlation between GABA and neural activation difference before and after a high frequency visual stimulus, an effect thought to reflect visual plasticity. In agreement with other studies, when only baseline visual stimulation-related activation was used, a negative correlation was found with GABA levels. However, results for this analysis were not published and only mentioned in the discussion. Schallmo et al. (19) used a spatial suppression paradigm where participants had to indicate the direction of moving gratings with varying contrasts. No correlations between GABA levels in the early visual cortex and the more laterally located visual motion complex and neural response in the same regions were found.

Glutamate levels repeatedly did not show a relationship to co-localised neural activity in studies of the occipital lobe (9, 12–14, 20, 21). Some of these studies utilised the same sequence to measure GABA and glutamate levels simultaneously (9, 12–14, 21). Two studies reporting a relationship with GABA levels found no association between neural activity and co-localised Glx (9, 12). Wijtenburg et al. (17) found a relationship between glutamate levels and an activation contrast comparing neural response before and after a high frequency visual stimulus. This relationship was also a positive one, similar to what had been observed with GABA levels. However, in this case, the association between glutamate levels and activity related to baseline visual stimuli was not reported. Finally, within the visual motion complex glutamate levels were positively correlated with neural response during motion perception in a spatial suppression paradigm (21).

#### Sensorimotor Cortex

Studies sampling metabolite levels in the sensorimotor cortex and adjacent areas (Table 2) mostly utilised a simple paradigm, in this case some form of finger-tapping task. In multiple studies, no significant associations were found between sensorimotor cortex GABA levels and local neural activity (15, 22–24). Using the same finger-tapping task, Draper et al. (24) also found no correlation between GABA levels in a supplementary motor area and BOLD %SC. Only one study (25), using a visually cued reaction time task, indicated a negative relationship between BOLD and GABA concentrations in the sensorimotor cortex. This same study was the only one to look at neural activity with glutamate levels in the sensorimotor cortex area and found no correlated activation clusters in a whole-brain regression.

**Table 2:** Sensorimotor cortex ^1^H-MRS studies.

**Authors**	***n*** **Male %** **Age M(SD)**	**System** **Sequence**	**Reference**	**fMRI task/stimuli**	**Relationship**	**Covariates**
**GABA**						
*Sensorimotor cortex*						
Bhattacharyya et al. (22) Bhattacharyya et al. (23)	9 11.1 38.3 (14.1)	3T MEGA-PRESS	Absolute	Bilateral finger tapping	Finger tapping (#) GABA	–
Draper et al. (24)	14 92.86 15.8 (3.2)	7T STEAM	/NAA	Finger-thumb opposition task	Finger tapping (#) GABA	–
Harris et al. (15)	18 38.9 27.8 (4.0)	3T MEGA-PRESS	/Wt, /Cr	Finger tapping	Finger tapping (#) GABA+	–
Stagg et al. (25)	12 50 23	3T MEGA-PRESS	/NAA	Visually cued reaction time task	Movement performance (–) GABA	–
*Supplementary motor area*						
Draper et al. (24)	14 92.86 15.8 (3.2)	7T STEAM	/NAA	Finger-thumb opposition task	Finger tapping (#) GABA	–
**Glutamate**						
*Primary motor cortex*						
Stagg et al. (25)	12 50 23	3T MEGA-PRESS	/NAA	Visually cued reaction time task	Motor performance (#) Glx	–

*Wt, Water; Cr, Creatine; NAA, N-acetylaspartate; (+) positive association; (#) no association; (–) negative association; GABA+, GABA + macromolecule; Glx, glutamate + glutamine*.

#### Anterior Cingulate Cortex/Medial Prefrontal Cortex

##### GABA Concentrations in the Anterior Cingulate Cortex/Medial Prefrontal Cortex

Due to the involvement of the anterior cingulate cortex (ACC) and medial prefrontal cortex (mPFC) in different processes such as emotion, attention and cognition (26), various task contexts have been used (Table 3). Many studies investigated NBR within this region, due to the close coupling between the ACC/mPFC and the default mode network (DMN). Four studies investigating GABA levels in the mPFC/ACC region found a positive relationship with regional NBR, i.e., a negative relationship with neural activity (27, 30, 31, 35). Northoff et al. (35) found that during conditions that produced little NBR compared to rest, this negative relationship between GABA levels and neural activity was not apparent. In agreement with these findings, using facial expressions Stan et al. (29) observed that mPFC GABA+ concentration was negatively correlated with subgenual ACC (sgACC) fMRI response to sadness. However, neural activity during other emotion categories—happy, fear, or anger—was not correlated with GABA+ concentrations. When using an emotion processing task, Levar et al. (32) only found a negative correlation with GABA+ levels when contrasting neural activity during negative emotional stimuli with response to positive emotional stimuli. Other studies failed to find negative correlations between regional GABA concentrations and neural activity, or positive relationships with NBR, during cognitive control tasks (34, 36), a verbal working memory task (5), a reward-guided decision-making task (28), and a fear conditioning, extinction, and extinction retrieval task (33).

**Table 3:** Medial prefrontal cortex/anterior cingulate cortex ^1^H-MRS GABA studies.

**Authors**	**n** **Male %** **Age M(SD)**	**System** **Sequence**	**Reference**	**fMRI task/stimuli**	**Relationship**	**Covariates**
*Medial prefrontal cortex*/*pregenual anterior cingulate cortex*						
Chen et al. (27)	19 31.6 24 (2)	3T MEGA-PRESS	/Wt	Working memory task (Sternberg item recognition paradigm)	High cognitive load NBR (+) GABA Low cognitive load NBR (#) GABA	Sex, co-localised Glu
Jocham et al. (28)	25 H 100 –	3T MEGA-PRESS	/Cr	Reward-guided decision-making task	Value difference BOLD signal (+) GABA Raw BOLD signal (#) GABA	–
Stan et al. (29)	16 50 22.42 (2.9)	3T MEGA-PRESS	/Cr	Implicit emotion processing and regulation task	Subgenual, pregenual, dorsal ACC: happy/fear/anger (#) GABA+ subgenual ACC: Sad (–) GABA+	–
Wiebking et al. (30)	9H 55.6 21.11 (2.9)	3T MEGA-PRESS	/NAA	Intero- & exteroceptive awareness paradigm	Interoceptive awareness NBR (#) GABA Exteroceptive awareness NBR (+) GABA	GM
Witt et al. (5)	20 40 17.4 (2.6)	3T MEGA-PRESS	/Wt	Verbal working memory task	Encoding NBR (#) GABA+ Recognition NBR (#) GABA+	–
Walter et al. (31)	24 25 34.6	3T 2D-JPRESS	/Cr	Emotional stimulation paradigm	Emotional stimulation NBR (+) GABA	Age
*Dorsal anterior cingulate cortex*						
Levar et al. (32)	70 100 21.8 (3.2)	3T MEGA-PRESS	/Cr	Fear conditioning and extinction task + extinction retrieval	Fear retrieval (#) GABA+ R amygdala: Fear retrieval (+) GABA+ R amygdala, cerebellum, middle cingulate gyrus, R insula: Late fear extinction (–) GABA+ Fear extinction retrieval (#) GABA+	–
Levar et al. (33)	68 100 21.7 (3.2)	3T MEGA-PRESS	/Cr	Emotion processing task	Hippocampus, cerebellum, R fusiform gyrus: all stimuli (+) GABA+ L amygdala: emotion > neutral (+) GABA+ Amygdala, L PCu, L middle occipital gyrus: positive > neutral (+) GABA+ L amygdala: negative > neutral (+) GABA+ R superior frontal gyrus: negative > neutral (–) GABA+ dACC, superior frontal gyrus, R MCC: negative > positive (–) GABA+	–
Overbeek et al. (34)	21 76.2 23.5 (4.5)	7T STEAM	/Wt	Stroop task	L postcentral gyrus, L inferior parietal lobe, L supramarginal gyrus, L angular gyrus, L middle occipital lobe, L middle frontal lobe, L inferior frontal lobe, L precentral gyrus, L superior temporal lobe, L rolandic operculum, L postcentral gyrus: cognitive control (–) GABA	Glu, Gln, WM
*Anterior cingulate cortex*						
Northoff et al. (35)	12 33.3 33.8	3T JD-PRESS	/Cr	Emotion processing task	All pictures NBR (+) GABA Picture judgement NBR (+) GABA Picture viewing NBR (#) GABA	Glx, GSH
Wang et al. (36)	25 0 27.5 (6.6)	3T MEGA-PRESS		Cognitive control hybrid task	Fronto-striatal regions: cognitive control (#) GABA	–

*Wt, Water; Cr, Creatine; NAA, N-acetylaspartic acid; NBR, negative BOLD response; (+) positive association; (#) no association; (–) negative association; GABA+, GABA + macromolecule; Glx, glutamate+glutamine; GSH, glutathione; PCu, Precuneus; dACC, dorsal anterior cingulate cortex; MCC, middle cingulate cortex; WM, white mater volume fraction within ^1^H-MRS voxel; GM, grey mater volume fraction within ^1^H-MRS voxel; /, OR. Neural activity is co-localised with the ^1^H-MRS sampling region unless otherwise specified in the relationship column [i.e., Region: task related activity (+/#–) GABA/Glu/Glx]*.

Correlations between GABA levels and distal neural activity were also found in some of these studies (32–34). Neural activity related to fear recovery in the amygdala was positively correlated with dACC GABA levels, while neural activity related to late phases of fear extinction in the right amygdala, cerebellum, middle cingulate gyrus, and right insula were negatively correlated with GABA (33). Levar et al. (32), using non-facial picture stimuli, found that dACC GABA concentrations correlated positively with activity in the left amygdala during positive emotion, negative emotion, all emotion and positive over negative emotion. Right amygdala activity related to positive emotion was also positively correlated with dACC GABA levels. Activity of the right superior frontal gyrus during negative pictures was negatively correlated with GABA levels. BOLD signal related to all picture conditions was positively correlated with GABA levels in the left and right hippocampi. ACC GABA concentrations were negatively associated with cognitive control-related BOLD signal in two clusters. The first was localised mainly to the left parietal cortex, spanning the postcentral gyrus, inferior parietal lobe, supramarginal gyrus, angular gyrus, and the middle occipital lobe. The second cluster was found mainly in the left dlPFC, including regions within the middle frontal lobe, inferior frontal lobe, precentral gyrus, superior temporal lobe, rolandic operculum, and postcentral gyrus.

##### Glutamate Concentrations in the Anterior Cingulate Cortex/Medial Prefrontal Cortex

Twenty-one studies sampled glutamate levels within the mPFC/ACC regions and investigated its relationship to neural activity within the same region as well as in other brain regions. Of those, 15 studies did not find an association between mPFC/ACC glutamate levels and local activation. These studies used a variety of tasks encompassing: emotion (31, 32), cognitive control (34, 37–40), working memory (5, 27), reward (28, 41), intero- and exteroceptive awareness (30), aversion (42), and a verbal fluency task (43). Nonetheless, other studies did find relation between glutamate concentration and regional neural activity. Stan et al. (29) found mPFC glutamate levels were correlated with pregenual ACC (pgACC) response to anger, but not with regional activity during viewing of other emotional categories. Using an empathy task, Duncan et al. (44) sought to investigate the relationship between pgACC glutamate levels and neural activity in the task-activated supragenual ACC, as well as between supragenual ACC glutamate with pgACC activity. A whole-brain regression with pgACC Glx levels showed a relationship to empathy-related BOLD in the supragenual ACC (a cluster overlapping with the supragenual ACC ^1^H-MRS voxel). supragenual ACC Glx levels, however, were not associated with pgACC activation during the empathy task. ACC glutamate levels were also studied in conjunction to neural activity during a salience processing task using emotional and erotic pictures (45). Activation in the sgACC related to unexpectedness of emotional pictures displayed a negative correlation with glutamate. Dissecting this association, the authors found that a positive relationship between glutamate and expected emotional picture viewing drove this finding. In a separate study, Cadena et al. (46) scanned a control group twice with a 6-week interval (46). ACC Glx levels were positively correlated with ACC activation during cognitive control at the baseline scan, a relationship that was not observed at the second scanning session 6 weeks later. Enzi et al. (47) sampled Glx concentrations from the pgACC, where they found a partial positive correlation to NBR during anticipation of reward and of no outcome. Through further investigation, the authors found that Glx concentrations were correlated to the rest condition and hypothesised that this relationship was largely driving the relationship between glutamate levels and deactivation during reward anticipation.

In terms of associations between glutamate levels and neural activity outside of the ^1^H-MRS region, three studies reported positive correlations with neural activity outside of the ACC/mPFC. Duncan et al. (42) found mPFC glutamate levels were correlated positively with sensorimotor cortex and left insula activation during the anticipation of aversion. In a different study, Duncan et al. (44) showed, through whole-brain regression with Glx levels in the pgACC, a significant correlation with empathy-related activation in the left precuneus, bilateral amygdala and putamen, right superior frontal gyrus and right supramarginal gyrus. Cadena et al. (46) observed a correlation with cognitive control-related activation within the insula, hippocampus, inferior parietal lobule and precuneus. Notably, the findings in the bilateral insula and left inferior parietal lobule were replicated in the sample 6 weeks later. Interestingly, one study observed different correlations depending on task load. Falkenberg et al. (39) found three activation clusters where there was a significant interaction between glutamate levels and the parameters of a cognitive control task: five auditory intensity difference conditions and the instruction of which ear to pay attention to. *Post-hoc* tests showed that in individuals with lower levels of glutamate, neural response was higher during high cognitive control conditions, i.e., when attention was directed towards the less salient stimulus, whereas in individuals with high levels of glutamate, neural activity was higher during low cognitive control conditions. Four studies found negative correlations between glutamate or Glx levels and neural activity outside of the ACC/mPFC. Overbeek et al. (34) observed ACC glutamate levels were positively associated with NBR in a cluster spanning from the PCC and precuneus to the occipital cortex and cerebellum. Von During et al. (45) also found that ACC glutamate levels were positively associated with NBR in the PCC, with higher glutamate predicting higher deactivation of this area in response to a variety of stimuli. Neural activity in a variety of other regions were also negatively correlated with ACC glutamate levels in this study (see Table 4). One study investigated ACC glutamate levels with cognitive control-related activation within the striatum only, to which a negative relationship was found in a composite group of ADHD patients, unaffected siblings and healthy controls (48). Similarly, Gleich et al. (41) found a negative relationship with ventral striatum activation in adolescents but not in adults.

**Table 4:** Medial prefrontal cortex/anterior cingulate cortex ^1^H-MRS glutamate studies.

**Authors**	***n*** **Male %** **Age M(SD)**	**System** **Sequence**	**Reference**	**fMRI task/stimuli**	**Relationship**	**Covariates**
*Medial prefrontal cortex*/*rostral anterior cingulate cortex*						
Brennan et al. (40)	28 46.4 32.4 (12.1)	3T 2D-JPRESS	/Cr	Emotional Stroop task	Cognitive control (#) Glu	Age, sex
Chen et al. (27)	19 31.6 24 (2)	3T Semi-LASER	/Wt	Working memory task (Sternberg item recognition paradigm)	Working memory (#) Glu	Sex, co-localised GABA
Duncan et al. (44)	13 30.8 31.6 (–)	3T PRESS	/Cr	Empathy task	supragenual ACC/MCC, L precuneus, amygdala/putamen, R superior temporal gyrus, R supramarginal gyrus: empathy (+) Glx	–
Duncan et al. (42)	12 50 23 (3.5)	3T MEGA-PRESS	/NAA	Aversion Task	Sensorimotor cortex, L posterior supramarginal gyrus, L Insula/operculum: anticipation of certain aversion (+^∧^) Glu Anticipation of certain aversion (#) Glx Anticipation of uncertain aversion (#) Glu/Glx	-
Enzi et al. (47)	19 52.6 29.6 (–)	3T PRESS	/Cr	Modified monetary incentive delay task	Fixation (+) Glx Reward anticipation (#) Glx Punishment anticipation (#) Glx No outcome (#) Glx Fixation > Reward anticipation (+) Glx Fixation > Punishment anticipation (#) Glx Fixation > No outcome (+) Glx	Age
Jocham et al. (28)	25 100 –	3T MEGA-PRESS	/Cr	Reward-guided decision-making task	Value difference BOLD signal (+) Glu Raw BOLD signal (#) Glu	–
Naaijen et al. (48)	32 68.7 22 (3.8), 21 (4.9), 20.7 (2.5)	3T PRESS	/Cr	Stroop task	Striatum: cognitive control (–) Glu	–
Stan et al. (29)	18 50 22.79 (3.04)	3T PRESS	/Cr	Implicit emotion processing and regulation task	Happy/sad/fear (#) pre-task Glu Anger (+) pre-task Glu	Age
					Happy/fear/anger/sad (#) post-task Glu	Co-localised GABA+
Walter et al. (31)	24 25 34.6	3T 2D-JPRESS	/Cr	Emotional stimulation paradigm	Emotional stimulation NBR (#) Glu	Age
Wiebking et al. (30)	18 50 17.4 (2.6)	3T MEGA-PRESS	/NAA	Intero- & exteroceptive awareness paradigm	Interoceptive/exteroceptive awareness NBR (#) Glx	GM
Witt et al. (5)	20 40 17.7 (2.6)	3T MEGA-PRESS	/Wt	Verbal working memory task	Encoding/recognition (#) Glx	–
*Dorsal anterior cingulate cortex*						
Cadena et al. (46)	20 70 33.0 (9.3)	3T PRESS	/Cr	Stroop task (baseline)	Insula, R ACC: cognitive control (+) Glx Hippocampus, inferior parietal, precuneus: cognitive control (+) Glx	–
	19			Stroop task (6 weeks later)	Insula: cognitive control (+) Glx L inferior parietal: cognitive control (+) Glx	–
Duncan et al. (44)	13 30.8 31.6 (–)	3T PRESS	/Cr	Empathy task	Empathy (#) Glx	–
Levar et al. (33)	68 100 21.7 (3.2)	3T MEGA-PRESS	/Cr	Emotion processing task	All stimuli/positive>neutral/negative>neutral/negative>positive//emotion>neutral (#) Glx	–
Overbeek et al. (34)	21 76.2 23.5 (4.5)	7T STEAM	/Wt	Stroop task	PCu, calcarine sulcus, cuneus, lingual gyrus, middle cingulum, posterior cingulum, cerebellum: cognitive control (–) Glu	GABA, Gln, WM
Reid et al. (38)	18 61.1 36.8 (12.0)	3T PRESS	/Cr	Stroop task	Cognitive control (#) Glx	–
Von During et al. (45)	27 65 29.8 (7.8)	7T STEAM	/Cr	Salience processing	R PCC/PCu, R occipital, cerebellum: all picture viewing (–) Glu PCC: erotic picture viewing/unexpected emotional picture viewing (–) Glu Emotional picture viewing (#) Glu R vStr, sgACC: expected emotional picture viewing (+) Glu	Age, sex, GM
Yücel et al. (37)	24 54.2 29.6 (6.5)	3T PRESS	Absolute	Multi-source interference task	Cognitive control (#) Glx	
*Anterior cingulate cortex*						
Falkenberg et al. (39)	40 50 m 25.0 (3.6), f 26.0 (4.0)	3T PRESS	/Cr	Dichotic listening task	ACC: cognitive control (#) Glu Basal ganglia, R orbitofrontal cortex, L inferior parietal lobe: high cognitive control (–) Glu Basal ganglia, R orbitofrontal cortex, L inferior parietal lobe: low cognitive control (+) Glu	
Fusar-Poli et al. (43)	17 58.8 25.5 (3.6)	3T PRESS		Verbal fluency task	Verbal fluency (#) Glu	–
Gleich et al. (41)	26 adults 46.2 26.1 (4.4)	3T PRESS		Virtual slot machine	vStr,ACC: win>loss (#) Glu	–
	28 adolescents 53.6 14.4 (0.6)	3T PRESS		Virtual slot machine	vStr: win>loss (–) Glu ACC: win>loss (#) Glu	–
Modinos et al. (49)	21 low schizotypy 57.1 27.0 (5.6)	3T PRESS	/Wt	Emotional viewing task	Emotional viewing (#) Glu	–
Northoff et al. (35)	12 33.3 33.8	3T 2D-JPRESS	/Cr	Emotion processing task	Emotion processing (#) Glx	–

*Wt, Water; Cr, Creatine; NAA, N-acetylaspartic acid; NBR, negative BOLD response; ACC, anterior cingulate cortex; MCC, middle cingulate cortex; vStr, ventral striatum; PCu, precuneus; PCC, posterior cingulate cortex; sgACC, subgenual ACC; Glx, glutamate+glutamine; WM, white mater volume fraction within ^1^H-MRS voxel; GM, grey mater volume fraction within ^1^H-MRS voxel; ^∧^, uncorrected finding; (+) positive association; (#) no association; (–) negative association; /= OR. Neural activity is co-localised with the ^1^H-MRS sampling region unless otherwise specified in the relationship column [i.e., Region: task related activity (+/#/–) GABA/Glu/Glx]*.

#### Dorsolateral Prefrontal Cortex

All studies sampling GABA or glutamate levels from the dorsolateral prefrontal cortex (dlPFC) used a working memory fMRI task (Table 5). Only one study, comparing children and adolescents with sport-related injuries not involving the head and with no history of concussion, to a group of concussed individuals, found a significant positive correlation between dlPFC GABA levels and local activation (50). Two studies did not find a relationship (15, 27). Similarly, three studies that investigated glutamate levels also found no relationships to regional neural activity (27, 51, 52).

**Table 5:** Dorsolateral prefrontal cortex ^1^H-MRS studies.

**Authors**	**n** **Male %** **Age M(SD)**	**System** **Sequence**	**Reference**	**fMRI task/stimuli**	**Relationship**	**Covariates**
**GABA**						
Harris et al. (15)	18 38.9 27.8 (4.0)	3T MEGA-PRESS	/Wt, /Cr	Working memory task (n-back task)	Working memory (#) GABA+	–
Friedman et al. (50)	10 50 15.2 (1.2)	3T MEGA-PRESS	/tCr	Working memory task (1-back task)	Working memory (+) GABA	–
Chen et al. (27)	19 31.6 24 (2)	3T MEGA-PRESS	/Wt	Working memory task (Sternberg item recognition paradigm)	Working memory (#) GABA	Sex, co-localised Glu
**Glutamate**						
Moon and Joeng (51)	18 61.1 30.7 (7.5)	3T PRESS	/Cr	Working memory task with face distractors	Working memory (#) Glx	
Chen et al. (27)	19 31.6 24 (2)	3T Semi-LASER	/Wt	Working memory task (Sternberg item recognition paradigm)	Working memory (#) Glu	Sex, co-localised GABA
Kaminski et al. (52)	35 H 82.9 34.3 (8.5)	3T PRESS	/Wt	Working memory task (n-back task)	Working memory (#) Glu	age

*Wt, water; Cr, creatine; tCr, creatine+phosphocreatine; Glx, glutamate+glutamine*.

#### Temporal Lobe

Within studies of the temporal lobe (Table 6), the auditory cortex, anterior temporal lobe and hippocampus were sampled for metabolites and fMRI tasks were largely based on the respective functions of the ^1^H-MRS sampling regions. Two studies found a negative relationship between GABA levels and local activation during thought retrieval and though suppression (54) and during semantic processing (53). However, Harris et al. (15) found no relationship between fMRI activity in the auditory cortex and local GABA levels.

**Table 6:** Temporal lobe ^1^H-MRS studies.

**Authors**	***n*** **Male %** **Age M(SD)**	**System** **Sequence**	**Reference**	**fMRI task/stimuli**	**Relationship**	**Covariates**
**GABA**						
*Auditory cortex*						
Harris et al. (15)	18 38.9 27.8 (4.0)	3T MEGA-PRESS	/Wt, /Cr	Auditory white noise	Auditory stimuli (#) GABA+	–
*Anterior temporal lobe*						
Jung et al. (53)	20 35 23 (4)	3T MEGA-PRESS	/NAA	Semantic association task	Semantic association (–) GABA	GM
*Hippocampus*						
Schmitz et al. (54)	30 23.3 24.7 (4.3)	3T 2D-JPRESS	/Cr	Thought suppression task (Think/No think task)	Think (–) GABA No-Think (–) GABA	Sex, GM, co-localised Glu, dlPFC GABA, functional control signal
**Glutamate**						
*Anterior temporal lobe*						
Jung et al. (53)	20 35 23 (4)	3T MEGA-PRESS	/NAA	Semantic association task	Semantic association (#) Glx	GM
*Hippocampus*						
Fusar-Poli et al. (43)	17 58.8 25.5 (3.6)	3T PRESS		Verbal fluency task	Verbal fluency (#) Glu	–
Valli et al. (55)	14 42.9 25.62 (3.7)	3T PRESS	/Wt	Episodic memory task	Parahippocampus: Memory encoding (+) Glu	–
Hutcheson et al. (56)	28 60.7 35.6 (11.1)	3T PRESS	/Cr	Episodic memory task	Encoding/Retrieval (#) Glx IFG: retrieval (+) Glx	–
Bossong et al. (57)	19 52.6 25.8 (5.6)	3T PRESS		Monetary incentive delay task	vStr: Reward anticipation (+) Glx	–

*Wt, water; Cr, creatine; GABA+, GABA+macromolecules; NAA, N-acetylaspartic acid; GM, grey matter volume fraction within ^1^H-MRS voxel; dlPFC, dorsolateral prefrontal cortex; IFG, inferior frontal gyrus; vStr, ventral striatum; /, OR. Neural activity is co-localised with the ^1^H-MRS sampling region unless otherwise specified in the relationship column (i.e. Region: task related activity (+/#/–) GABA/Glu/Glx)*.

Unlike GABA, hippocampal glutamate levels were not found to be correlated with local activity in several studies (43, 55, 56). Glutamate levels were, however, positively correlated to neural activity outside of the hippocampus: parahippocampus (55), inferior frontal gyrus (56) and ventral striatum (57).

#### Insula

Three studies (30, 58, 59) measured relationships between metabolites and neural activity in this region (Table 7). Lipp et al. (58) reported a negative association between GABA concentration and insula neural activity only to animal picture stimuli, while pictures of other categories did not elicit such association. Interestingly, another study (30) identified a positive correlation between GABA levels and neural response to interoceptive awareness. Insula GABA levels were additionally correlated to distal neural activity: positively to activation in the amgydala and ventral striatum (58), and negatively to activation in the medial cingulate cortex and supplementary motor area (59). Meanwhile, glutamate levels did not correlate with neural activity in the insular cortex (30, 59).

**Table 7:** Insula ^1^H-MRS studies.

**Authors**	**n** **Male %** **Age M(SD)**	**System** **Sequence**	**Reference**	**fMRI task/stimuli**	**Relationship**	**Covariates**
**GABA**						
Wiebking et al. (30)	15 66.7 23.13 (4.58)	3T MEGA-PRESS	/NAA	Intero- & exteroceptive awareness paradigm	Interoceptive awareness (+) GABA Exteroceptive awareness (#) GABA	GM
Lipp et al. (58)	29 0	3T MEGA-PRESS	/Wt	Fear inducing paradigm	Whole brain: negative>neutral (#) GABA+ L amygdala, insula, vStr, frontal cortex: spiders>animals (+) GABA+ Neutral/negative/spiders (#) GABA+ Animals (–) GABA+	–
Cleve et al. (59)	27 100 24.9 (3.0)	3T MEGA-PRESS	/tCr	Pain stimulation	Pain perception (#) GABA+ MCC/SMA: pain perception (–) GABA+	–
**Glutamate**						
Wiebking et al. (30)	15 66.7 23.13 (4.58)	3T MEGA-PRESS	/NAA	Intero- & exteroceptive awareness paradigm	Interoceptive awareness (#) Glu/Glx Exteroceptive awareness (#) Glu/Glx	GM
Cleve et al. (59)	27 100 24.9 (3.0)	3T MEGA-PRESS	/tCr	Pain stimulation	Pain perception (#) Glx	–

*Wt, water; NAA, N-acetylaspartic acid; tCr, creatine+phosphocreatine; GM, grey matter volume within ^1^H-MRS voxel; vStr, ventral Striatum; MCC, middle cingulate cortex; SMA, supplementary motor area; /, OR. Neural activity is co-localised with the ^1^H-MRS sampling region unless otherwise specified in the relationship column (i.e. Region: task related activity (+/#/–) GABA/Glu/Glx)*.

#### Posterior Cingulate Cortex/Precuneus

Two studies investigated both GABA and glutamate levels within the posterior cingulate cortex (PCC)/precuneus (PCu) region (18, 60) (Table 8) that typically deactivate during some cognitive tasks but activate putatively during processing of scenes to support autobiographical memory and imagined events (61). Deactivation of the PCC/PCu region as well as the entire DMN (including the mPFC, PCC/PCu, and bilateral parahippocampus) during a working memory task were investigated separately in relation to glutamate and GABA concentrations (60). Regression models showed that as glutamate increased NBR decreased (i.e., weaker deactivation). Conversely, GABA levels were positively correlated with NBR for all but the lowest cognitive load (1-back condition), i.e., as GABA levels increased, deactivation increased, in both the model with PCC/PCu deactivation and DMN deactivation as dependent variable. In contrast, when using a perceptual discrimination task, Costigan et al. (18) found no association between PCC/PCu activation and local Glx and GABA+ levels.

**Table 8:** Posterior cingulate cortex/precuneus ^1^H-MRS studies.

**Authors**	***n*** **Male %** **Age M(SD)**	**System** **Sequence**	**Reference**	**fMRI task/stimuli**	**Relationship**	**Covariates**
**GABA**						
Hu et al. (60)	24 58.3 34.4 (8.6)	3T MEGA-PRESS	/Wt	Working memory task (n-back)	Low cognitive load NBR (#) GABA+ Mid/high cognitive load NBR (+) GABA+ DMN: low cognitive load NBR (#) GABA+ DMN: mid/high cognitive load NBR (+) GABA+	Age, GM, co-localised Glu
Costigan et al. (18)	40 30 22.1 (2.1)	3T MEGA-PRESS	/Wt	Odd-one-out task	Scene discrimination (#) GABA+	–
**Glutamate**						
Hu et al. (60)	24 58.3 34.4 (8.6)	3T MEGA-PRESS	/Wt	Working memory task (n-back)	All cognitive loads NBR (–) Glu DMN: low/mid cognitive load NBR (–) Glu DMN: high cognitive load NBR (#) Glu	Age, GM, co-localised GABA
Costigan et al. (18)	40 30 22.1 (2.1)	3T MEGA-PRESS	/Wt	Odd-one-out task	Scene discrimination (#) Glx	–

*Wt, water; GM, grey matter volume within ^1^H-MRS voxel; Glx, glutamate+glutamine; NBR, negative BOLD response; DMN, default mode network; /, OR. Neural activity is co-localised with the ^1^H-MRS sampling region unless otherwise specified in the relationship column (i.e. Region: task related activity (+/#/–) GABA/Glu/Glx)*.

#### Other Regions

A few studies have sampled GABA and glutamate levels from less frequently inspected regions combined with neural activity (Table 9). Harris et al. (15) found no relationship between activation related to eye saccades and GABA levels in the frontal eye field. Two studies sampled glutamate from the thalamus, both using a verbal fluency task. Fusar-Poli et al. (43) found no significant effects, while Allen et al. (64) found significant correlations with activation in the right superior frontal gyrus/sulcus and the right cingulate sulcus. Two studies found a positive correlation between Glx levels and local activity in two different brain regions: the substantia nigra (63) and the dorsal striatum (62).

**Table 9:** Remaining ^1^H-MRS studies.

**Authors**	***n*** **Male %** **Age M(SD)**	**System Sequence**	**Reference**	**fMRI task/stimuli**	**Relationship**	**Covariates**
**GABA**						
*Frontal eye field*						
Harris et al. (15)	18 38.9 27.8 (4.0)	3T MEGA-PRESS	/Wt, /Cr	Eye saccades	Saccades (#) GABA+	–
**Glutamate**						
*Thalamus*						
Allen et al. (2015)	27 66.7 24.4 (4.6)	3T PRESS		Verbal fluency task	R superior frontal gyrus/sulcus, R cingulate sulcus: verbal fluency (+) Glx	–
Fusar-Poli et al. (43)	17 58.8 25.5 (3.6)	3T PRESS		Verbal fluency task	Verbal fluency (#) Glu	–
*Dorsal striatum*						
Lorenz et al. (62)	32 59.4 47.3 (19.3)	3T PRESS	/Wt	Modified stop signal task	L caudate nucleus: response inhibition (+) Glx	–
*Substantia nigra*						
White et al. (63)	19 57.9 36.5 (12.1)	3T PRESS	/Cr	Monetary reward decision task	vStr/NAc: prediction error (#) Glx substantia nigra: prediction error (+) Glx	–

*Wt, water; /, OR; GABA+, GABA+macromolecules; Glx, glutamate+glutamaine; vStr, ventral striatum; NAc, nucleus accumbens. Neural activity is co-localised with the ^1^H-MRS sampling region unless otherwise specified in the relationship column (i.e. Region: task related activity (+/#/–) GABA/Glu/Glx)*.

### Meta-Analysis of Associations With GABA Concentrations

**Figure 2:**
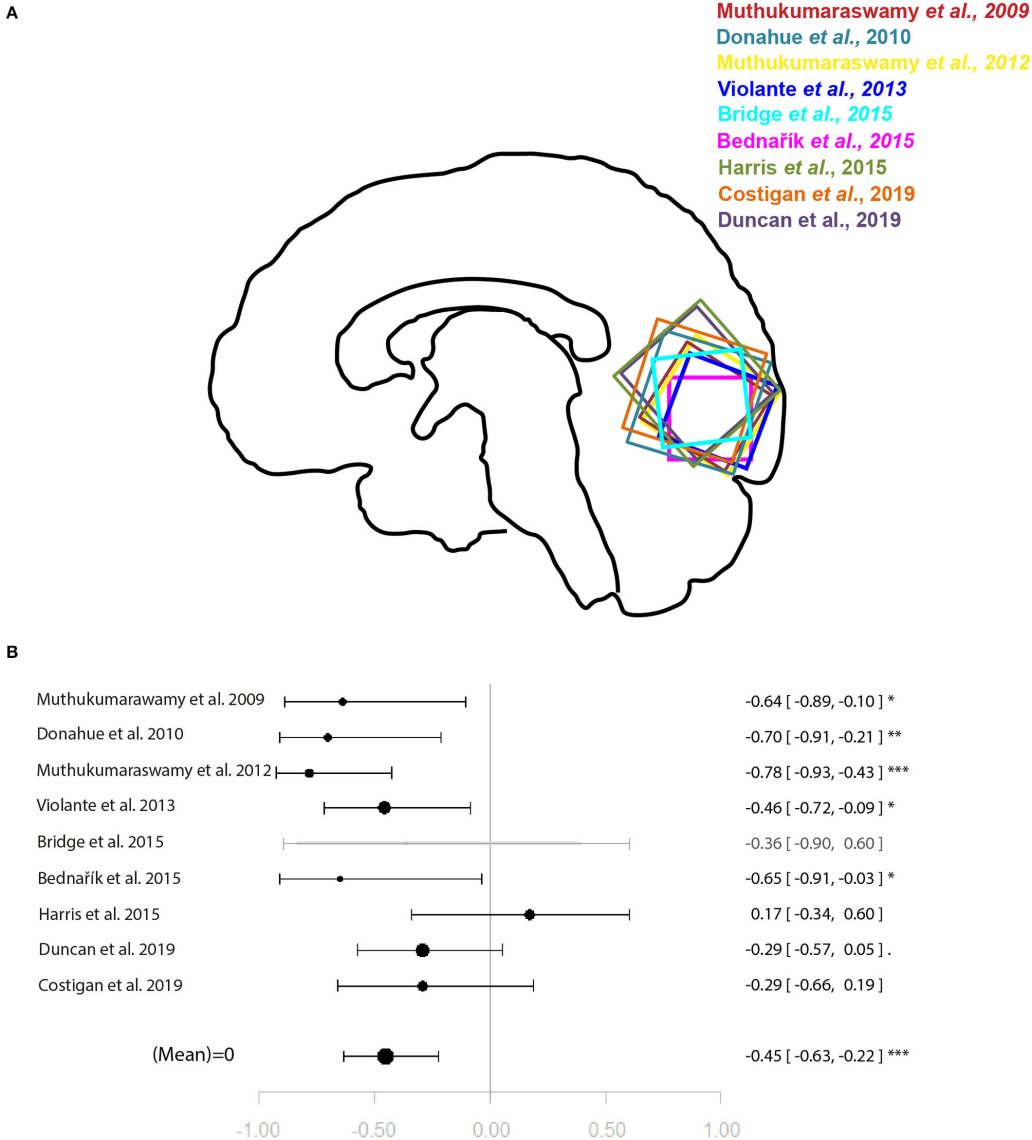
Meta-analysis of studies investigating occipital lobe GABA levels and visual perception-related neural activity in the occipital lobe. **(A)** Position of occipital voxels per study. **(B)** Forest plot of meta-analysis results. *p* < 0.10; ^*^p < 0.05; ^**^p < 0.01; ^***^p < 0.001.

**Figure 3:**
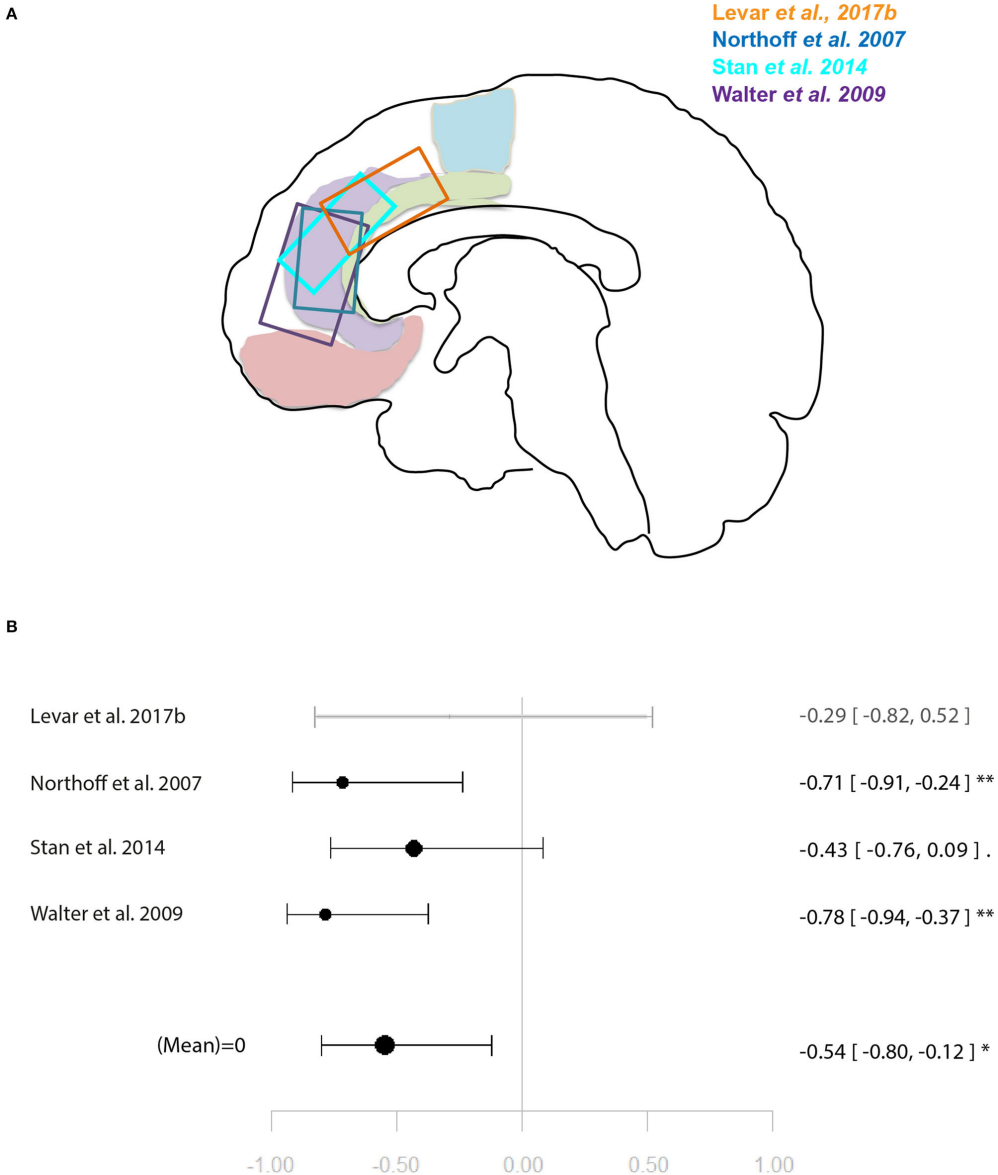
Meta-analysis of studies investigating GABA levels and emotion related neural activity in the anterior cingulate cortex. **(A)** Position of ACC voxels per study. **(B)** Forest plot of meta-analysis results. *p* < 0.10; ^*^p < 0.05; ^**^p < 0.01.

Within the studies sampling GABA, two groups of more than four studies emerged: occipital lobe GABA levels in combination with a visual task, and ACC GABA levels in combination with emotion processing tasks. For illustration of the voxel positions of each study per meta-analysis see Figures 2, 3.

For the relationship between GABA levels and BOLD within the occipital lobe, the systematic search for articles identified nine eligible studies for quantitative analysis (9–16, 18). The results of the meta-analysis demonstrated a significant relationship between GABA and BOLD in the occipital lobe (*r* = −0.45 [−0.63, −0.22], *p* = 0.002, *I*^2^ = 47.06%) (Figure 2).

Another four studies investigated ACC GABA in relation to local response to emotion tasks (29, 31, 32, 35). Correlation coefficients were available for three of these, while Levar et al. (32) used a regression with ACC GABA levels as a covariate. The latter found a negatively correlated cluster in the dorsal ACC for negative vs. positive emotion, however, this result was not used for the main meta-analysis for which comparable contrasts were selected only (specific or all emotion vs. baseline). The main meta-analysis of this relationship yielded a significant negative relationship between ACC GABA and regional emotion-related neural activity (*r* = 0.53 [−0.79, −0.11], *p* = 0.02, *I*^2^ = 34.04%) (Figure 3).

### Meta-Analysis of Associations With Glutamate Concentrations

**Figure 4:**
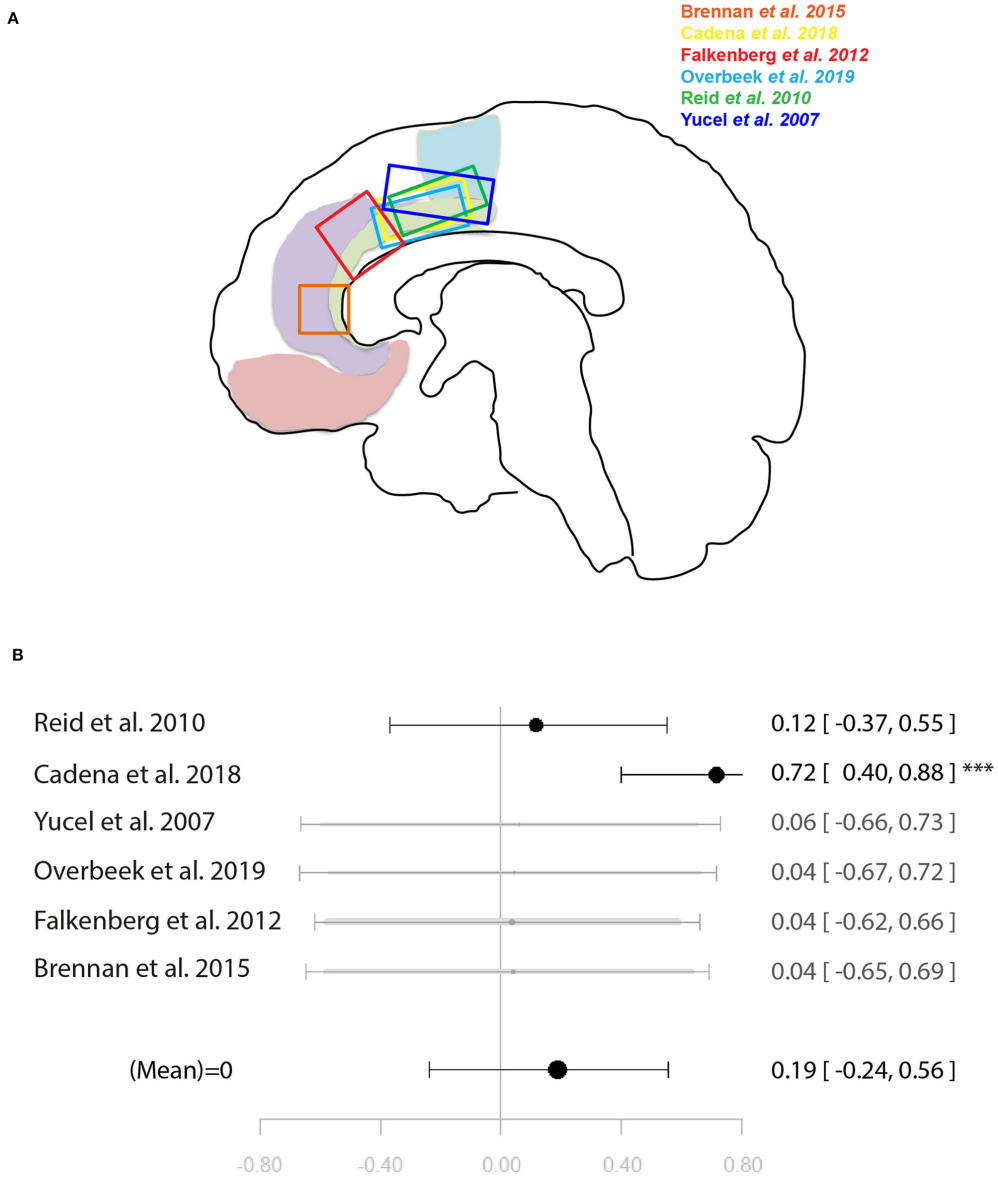
Meta-analysis of studies investigating ACC glutamate levels and regional cognitive control-related neural activity in the anterior cingulate cortex. **(A)** Position of ACC voxels per study. **(B)** Forest plot of meta-analysis results. *p* < 0.10; ^***^p < 0.001.

**Figure 5:**
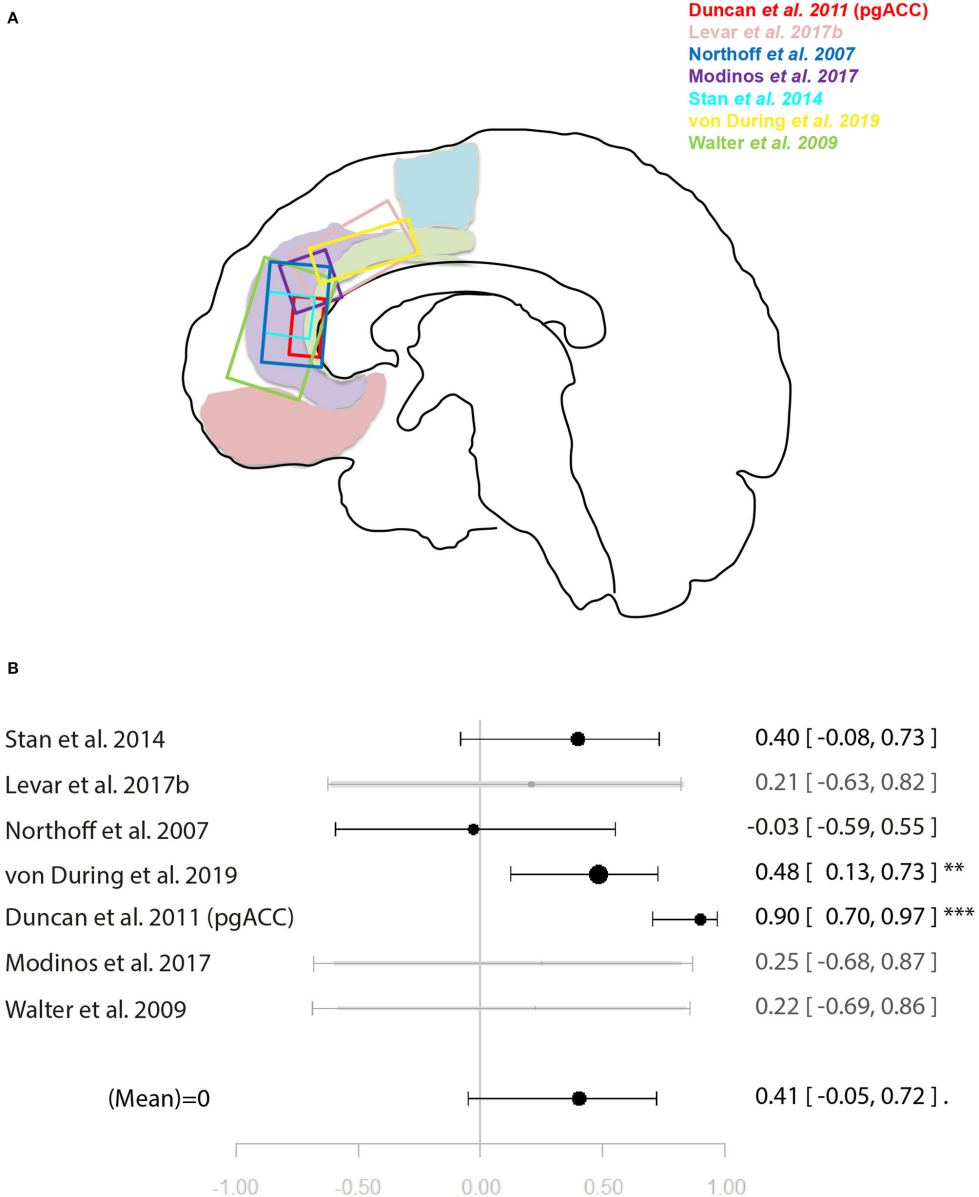
Meta-analysis of studies investigating ACC glutamate levels and regional emotion related neural activity in the anterior cingulate cortex. **(A)** Position of ACC voxels per study. **(B)** Forest plot of meta-analysis results. *p* < 0.10; ^**^p < 0.01; ^***^p < 0.001.

Two meta-analyses were performed. One combined glutamate levels in the ACC and local activation during cognitive control tasks. The other combined glutamate levels in the ACC and local activation during emotional tasks. For illustration of the voxel positions of each study per meta-analysis see Figures 4, 5.

Six studies (34, 37–40, 46) were included in the meta-analysis of the relationship between ACC glutamate levels and local activation during cognitive control. These were a mixture of studies using correlation and regression analysis. When regression analysis had been performed on the whole-brain level and no significant correlation with the ACC was found, this was entered into the meta-analysis as a non-significant result to avoid bias. The meta-analysis did not find support for a relationship between ACC glutamate levels and neural activity during cognitive control [*r* = 0.19 (−0.24, 0.56), *p* = 0.39, *I*^2^ = 71.62] (Figure 4).

For the meta-analysis investigating the relationship between ACC glutamate levels and local activation during emotion processing, seven studies were included (29, 31, 32, 35, 44, 45, 49). As with the previous meta-analysis on cognitive control, studies utilised a mixture of correlation and regression analyses. This analysis marginally missed significance for a positive relationship between ACC glutamate levels and regional emotion related neural activity [*r* = 0.41 (−0.04, 0.72), *p* = 0.07, *I*^2^ = 79.69%] (Figure 5).

## Discussion

The main finding of this meta-analysis was that GABA levels were negatively correlated with co-localised neural activity within the ACC during emotion processing and within the occipital lobe during visual stimulation. Further meta-analyses showed that glutamate levels within the ACC were not correlated with co-localised neural activity during either cognitive control or emotion processing, however, the latter meta-analysis narrowly missed significance. These findings were further supported by a systematic review of all fMRI-^1^H-MRS association studies to date, where increasing resting GABA levels were repeatedly associated with either weaker neural activation or stronger neural deactivation. On the other hand, glutamate levels were largely not correlated with intra-regional neural activity, although a handful of studies did find a positive correlation. An additional finding was that glutamate concentrations were largely positively correlated with regions outside of the ^1^H-MRS sampling area; however, negative inter-regional associations were also identified in multiple studies. GABA concentrations were less likely to be associated with regions outside of the ^1^H-MRS sampling area with only a few studies providing evidence of a positive or negative relationship with inter-regional neural activation depending on the metabolite sampling region and the neural activation region.

These findings support and extend prior reported trends. Previously, in a narrative review Duncan et al. (4) highlighted that GABA tended to be negatively correlated with regional neural activity in the visual, auditory, sensorimotor and anterior cingulate cortices, while glutamate was positively correlated with neural activity in regions outside of the sampling region of the metabolite. In the present study, by using a systematic rather than narrative review method and capitalising on the large increase in available studies specifically using ^1^H-MRS and fMRI, a more region-dependent relationship between these specific measures emerged. The negative relationship between GABA levels and regional neural activation was confirmed within the occipital cortex, ACC/mPFC, temporal lobe, and PCC. However, regions such as the sensorimotor cortex, auditory cortex and dorsolateral prefrontal cortex either did not exhibit this relationship clearly or in a few studies the relationship was in the opposite direction (30, 50). A negative relationship between GABA levels and regional neural activation appears intuitive: increased inhibition as measured through resting state ^1^H-MRS would be associated with decreased net activation of a region through regulation of resting excitatory activity (4, 65). Prior work in animals found that pharmacological manipulation increasing endogenous GABA concentrations produced lower positive BOLD signal changes (66). In the context of NBR, previous work in rats found hyperpolarisation of neurons through neuronal inhibition to correspond to a decrease in blood oxygenation (67). Findings of a positive relationship between GABA levels and neural activity may be a result of more complex neural processes that require suppression of neural response to irrelevant stimuli to promote concurrent activation to target stimuli (68). A relationship between GABA levels and neural activity in distal regions had not previously been identified in the literature (4). While initial studies suggested that the relationship between GABA levels and neural activity was limited to the same region (4, 68), a handful of recent studies reported both negative and positive relationships with activation outside of the ^1^H-MRS voxel (32–34, 58–60). Negative relationships with inter-regional neural activity may be the result of local inhibition of excitatory projections that regulate activity in other regions (32–34).

In terms of glutamate, a few studies observed a positive relationship between glutamate and local neural activity in the ACC and PCC (29, 44, 46, 60). Additionally, both positive and negative relationships with distal neural activity were found. These findings add to the simplified positive relationship with inter-regional neural activity reported in Duncan et al. (4). A negative relationship between BOLD activity in DMN regions, such as the PCC/PCu, were speculated to be due to higher ACC glutamate levels supporting facilitation of salience processing, thus resulting in stronger deactivation of DMN regions (39, 45). However, this relationship seems to be region-dependant as glutamate levels measured within the DMN, specifically the PCC, were positively related with DMN neural activation, where higher glutamate levels predicted lower local deactivation (60). The positive relationship between glutamate levels and intra-regional neural activity may stem from an excitatory drive stimulating neural activation within the same region through recurring feedback (46, 69). However, the relationship between glutamate levels and intra-regional neural activity may be difficult to replicate: one study failed to replicate a positive correlation between ACC glutamate and ACC neural activity at a 6-week follow-up scan within the same cohort (46). In fact, Enzi et al. (47) suggested resting glutamate levels may be more closely related to resting-state activation, thus being less intimately linked with task-related activity. As previously observed by Duncan et al. (4), glutamate levels were positively associated with neural activity outside of the sampling regions for glutamate. The long-range projections of glutamatergic neurons (70) are thought to influence neural activity in distal regions, thus giving rise to this relationship. However, a number of studies have shown that these projections may not simply reflect positive relationships with neural activity, but may also indicate that glutamate levels can negatively predict neural activity inter-regionally.

The ACC has been commonly investigated across studies, for which effects have been inconsistent. The discrepancies in ^1^H-MRS x fMRI relationships may arise from functional segregation within the ACC (71), its involvement in different behaviours (72), and that it hosts both task-positive and task-negative subdivisions (e.g., pgACC and supragenual ACC). The ability of ^1^H-MRS to discriminate between ACC subregions is still limited due to the ^1^H-MRS voxel size, especially in the case of GABA. This may be improved by the use of high-field MRI scanners that will allow for smaller voxel sizes (73). An additional consideration is that relationships between metabolite levels and neural activity may also be task-specific. Studies that used an active control task, rather than a low-level baseline condition such as a fixation cross [e.g., (44, 53, 54)], indicate that baseline metabolite levels are not simply correlated with any type of neural response. When neural response was confirmed for both the task of interest and the control task, different relationships between GABA levels and neural response were found indicating that metabolite levels may not be simply associated with all types of neural activity within a region (54).

Despite some incongruent findings, a pattern of neurochemical-neurofunctional relationships has emerged, which give valuable insight into psychiatric disorders where this relationship is aberrant. Measurement of metabolite levels at rest with ^1^H-MRS is thought to reflect individual differences in number of glutamatergic and GABAergic neurons, number of glutamatergic and GABAergic synapses, and glutamate and GABA turnover (68). It is conceivable, then, that abnormalities in metabolite levels would be reflected in differential neurochemical-neurofunctional relationships. In psychiatric disorders such as schizophrenia, case-control studies have suggested an aberrant relationship between ACC glutamate and local neural activity (46) and in regions receiving inputs from the ACC, such as the insula and DMN (34, 46, 74). Differences were found in the relationship between hippocampal Glx and inferior frontal gyrus activation, with medicated schizophrenia patients showing a less robust relationship (56) potentially reflecting differential coupling between the two regions (75). Activation related to prediction error within the substantia nigra was not correlated with regional glutamate levels in schizophrenia patients, which was interpreted as a glutamatergic dysfunction leading to abnormal prediction error coding (38). Furthermore, a relationship between glutamate and working memory activation, both within the dlPFC, was found in unmedicated schizophrenia patients where no such relationship existed in healthy controls—potentially due to compensatory action to glutamatergic insensitivity (52). Given the known and increasingly well-characterised abnormalities in glutamatergic and GABAergic function associated with this psychiatric disorder (76, 77) and the close relationship between metabolites and neurofunction, abnormalities in brain function should arise. The neurochemical-neurofunctional relationship has been investigated less frequently in other psychiatric disorders such as obsessive-compulsive disorder and major depressive disorder. While this line of investigation showed no differences in the relationship in patients with obsessive compulsive disorder (40, 51), patients with major depressive disorder showed stronger coupling between glutamate levels and NBR compared with healthy controls (31). Walter et al. (31) concluded that reduced resting state activity was driven by glutamatergic metabolism in patients with major depressive disorder. Differences in ^1^H-MRS x fMRI relationships may be the result of variations in neurochemistry, differential abnormal functions regionally and inter-regionally, as well as structural abnormalities. Despite the correlational nature of all the studies identified here, understanding healthy relationships between metabolite and neural activity allows some insight into compensatory action within mental illness and can provide potential pharmaceutical targets for intervention. Targeting GABAergic or glutamatergic neurotransmission may be a promising avenue to treating symptoms of schizophrenia (78) and major depressive disorder (79).

### Considerations on the Choice of Analytical Approaches

The studies included in this review used two main types of methods to derive relationships. Harris et al. (15) demonstrated that when using the correlational technique with %SC, the source of sampling for %SC could have a large effect on the observed relationship. Harris et al. (15) tested five different BOLD activation sources: peak within the ^1^H-MRS voxel, mean across the ^1^H-MRS voxel, mean across significant activation, peak within anatomical ROI and mean across anatomical ROI. From the peak within ^1^H-MRS voxel to the mean across significant activation, there was a maximum change in effect size of *r* = 0.47, although it should be noted that these individual correlation coefficients were not statistically significant due to Bonferroni correction. Despite these potential sources of variation, the vast majority of articles cited in our systematic review that used the correlational technique with %SC only tested the relationship between metabolites and neural activation with one source of %SC, which may cause inconsistencies in findings.

### Effects of Potential Confounders

Previously published studies controlled for potential confounds in diverse ways. Commonly used covariates were age, voxel tissue composition and sex. Age may have region-specific effects on levels of glutamate (80) and of GABA (81) and thus should be consistently controlled for. Our systematic review was not restricted to a certain age limit and instead attempted to collect data on all age ranges and noted whether studies controlled for age. Similarly, a potentially confounding effect of sex on metabolite levels was not consistently considered in individual studies, although sex has previously been identified to alter levels of glutamate (82) and GABA (83).

A further issue refers to the use of other regions as specificity control (4). In order to confirm that a significant association is not a global effect, metabolite analyses should be conducted both for the main region of interest as well as for a control region. Similarly, to ensure relationships can be specific to the experimental task, researchers should also evaluate the relationship between metabolite levels and a control, unrelated task. However, time constraints and participant fatigue during scanning may not enable such control measures in all studies.

### Future Directions

Although it is tempting to suggest that future studies using the correlational approach should test various sources of %SC [e.g., (15)], the multiple testing corrections needed would require strong relationship effects between metabolite and BOLD signal. However, the present systematic review and meta-analyses seem to suggest such effect sizes are moderate at best for the metabolite glutamate. An alternative suggestion would be to consistently utilise %SC derived from the peak voxel within the ^1^H-MRS voxel as many studies have done, with deviations from a standard source of %SC needing to be specifically addressed and justified.

Other future suggestions for studies of this kind are to include age and sex as confounding covariates more consistently. Similarly, task and region specificity controls should be included wherever possible. One reason for the lack of substantial conclusions to be made from the above listed studies is the heterogeneity of ^1^H-MRS locations, BOLD %SC sampling locations and region coverage within regression studies. Replication studies would allow more insight into studies that used emotional, working memory, reward and motor tasks. Additionally, combination with additional modalities such as arterial spin labelling as a different measure of brain function may help parse out the less consistent relationships seen here. Future studies should also aim to investigate the ^1^H-MRS x fMRI relationship in further psychiatric disorders that feature glutamatergic or GABAergic abnormalities, such as autism spectrum disorder (ASD). Abnormalities in excitation-inhibition balance are well-known and characterised in individuals in ASD (84, 85), where reduced neural activity in corticostriatal circuits have been proposed (86, 87) to be linked to the reduction of striatal glutamate (85). However, further investigation of these hypotheses and the nature of the neurochemical-neurofunctional relationship in ASD is required. Finally, capitalising on the findings presented here, future studies in psychiatric populations such as schizophrenia patients may modulate metabolite levels such as glutamate to test whether neurofunction can be modulated as a result.

## Conclusions

The significant expansion of studies combining multi-modal ^1^H-MRS and fMRI in recent years has enabled more detailed examination of the interactions between neurochemistry and neurophysiology in the healthy brain. These studies have shown robust negative correlations between GABA levels and local activation in regions such as the occipital cortex and ACC, and positive correlations between glutamate levels and activation in distal regions. These findings have implications for our understanding of pathophysiology of psychiatric disorders such as schizophrenia, a field in which studies combining ^1^H-MRS and fMRI are burgeoning in recent years. In this context, findings from multimodal studies may provide key insights into the neurobiology of schizophrenia not only by informing how the interplay between neurochemistry and neurophysiology may be altered prior and across disease stages, but also by their translational potential to elucidate whether pharmacological modulation of glutamate and/or GABA may be a new target for addressing abnormal neurophysiology in this psychiatric disorder.

## Data Availability Statement

The original contributions presented in the study are included in the article/Supplementary Material, further inquiries can be directed to the corresponding author/s.

## Author Contributions

GM, AK, and MJK: concept and design. CB and AK: systematic search. PBL and AK: data extraction. AK: statistical analysis and drafting of the manuscript. MJK and AK: statistical support. CD, JMS, PBL, GM, and AK: critical revisions of the manuscript. CD, JMS, and GM: supervision. All authors contributed to the article and approved the submitted version.

## Conflict of Interest

The authors declare that the research was conducted in the absence of any commercial or financial relationships that could be construed as a potential conflict of interest.
