# Supplementary material for: GABA, Glutamate and Neural Activity: A Systematic Review With Meta-Analysis of Multimodal 1H-MRS-fMRI Studies
Source: Front Psychiatry. 2021 Mar 8;12:644315. doi: 10.3389/fpsyt.2021.644315 (PMC7982484; doi:10.3389/fpsyt.2021.644315)
Supplement: Supplementary file 1 [file Data_Sheet_1.docx]

Supplementary Material

# Supplementary Methods

Table S1. Retrieval strategy terms for electronic literature search

| Database | Search Syntax |
| --- | --- |
| Pubmed | (Functional Imaging OR Functional Neuroimaging OR fMRI OR functional magnetic resonance imaging OR functional MRI) AND ((Magnetic Resonance Spectroscopy OR MRS OR Spectroscopy OR cortical) AND ((gamma-aminobutyric acid OR GABA) OR (glutamate OR Glutamic acid))) |
| Medline (Ovid) | (Functional imaging.mp. OR exp functional neuroimaging/ OR fMRI.mp. OR exp functional magnetic resonance imaging/ OR functional MRI.mp.) AND ((magnetic resonance spectroscopy.mp. OR exp nuclear magnetic resonance spectroscopy/ OR MRS.mp. OR spectroscopy.mp. OR cortical.mp.) AND ((gamma-aminobutyric acid.mp. OR GABA.mp. OR exp 4 aminobutyric acid/) OR (exp glutamic acid/ OR glutamate.mp))) |
| Embase (Ovid) |  |
| PsycINFO (Ovid) |  |

Table S2. Criteria for inclusion/exclusion of studies to review

| ***Inclusion Criteria*** |
| --- |
| *Study designs:* |
| - - Observational studies   - Or: Cohort studies   - Or: Case-control studies   - Or: Cross-sectional studies   - Or: Experimental studies |
| *Population of interest:* |
| - - Healthy human participants or healthy human control group |
| *Interventions or exposures:* |
| - - None   - Or: Baseline outcome measures have been conducted and reported |
| *Outcome of interests:* |
| - - Resting Glutamate or GABA metabolite levels measured by ^1^H-MRS   - Association between ^1^H-MRS measure and:     - Task-related fMRI BOLD response     - FC derived via seed-based analysis |
| ***Exclusion Criteria*** |
| - - Non-empirical articles: commentaries, reviews, editorial letters   - Meta-analyses   - Non-peer-reviewed articles   - Animal studies   - Not English   - Case-studies |

## Sensitivity analyses

Sensitivity analyses were performed when studies included in the meta-analysis had conducted more than one analysis for the outcome of interest. Methods for running these analyses are identical to the methods outlined in the main text (2.1 Meta-analysis). Significance was determined using two-tailed 95% confidence intervals.

To get the full range of variability, a maximum effect and a minimum effect sensitivity analysis was performed. For the maximum effect sensitivity analysis, the analysis result with the most positive correlation from each study was selected and entered into the meta-analysis. For the minimum effect sensitivity analysis, the result with the most negative correlation was entered into the meta-analysis.

# Supplementary results

## Methodological details of studies included in meta-analyses

Table S3 Methodological details of studies of occipital GABA and visual-related neural activity

| Study | fMRI paradigm | Stimuli | Contrast |
| --- | --- | --- | --- |
| Muthukumaraswamy et al. 2009 | Visual | 1.5-2s stationary vertical gratings / 10s fixation cross; 42 events | Grating > fixation cross |
| Donahue et al. 2010 | Visual | 20s flashing checkerboard / 40s rest; 3 blocks | Flashing checkerboard > rest |
| Muthukumaraswamy et al. 2012 | Visual | 1.5-2s stationary vertical gratings / 18s fixation cross; 45 events | Grating > fixation cross |
| Violante et al. 2013 | Visual | 1.5-2s moving grating / 10s fixation point; 30 events | Grating > fixation cross |
| Bridge et al. 2015 | Visual | Experiment 1: 120s diffuse light / 60s dark; 3 blocks  Experiment 2: 15s flickering checkerboard / 15s fixation cross | Experiment 1: diffuse light > dark  Experiment 2: checkerboard > fixation cross |
| Bednařik et al. 2015 | Visual | 7.5s flickering checkerboard / 20s rest; 8 blocks | Checkerboard > rest |
| Harris et al. 2015 | Visual | 1.5-2s stationary vertical gratings / 10s fixation cross; 42 events | Grating > fixation cross |
| Costigan et al. 2019 | Odd-one-out | Trial: 3 stimuli of one category: scenes, faces, objects or squares; 6s trial / 0.5-3s rest; 72 trials x 3 runs | Scenes > squares |
| Duncan et al. 2019 | Visual | 1.5-2s stationary vertical grating / 18-20s fixation cross; 46 trials | Grating > fixation cross |

Table S4 Methodological details of studies of ACC GABA and emotion-related neural activity

| Study | fMRI paradigm | Stimuli | Contrast |
| --- | --- | --- | --- |
| Levar et al. 2017b | Emotion processing | 20 negative / neutral / positive IAPS pictures; positive and negative matched for arousal; 4s stimuli / 3-5s fixation cross | Negative + positive > neutral |
| Northoff et al. 2007 | Emotional picture viewing and judgement | Positive / negative IAPS pictures; 4s picture viewing or picture judgement with or without 8-11.5s preceding expectancy period / 6-8s fixation cross; 159 trials | Negative + positive > fixation cross |
| Stan et al. 2014 | Implicit emotion processing and regulation | 1s movies of facial expressions morphing from neutral to happy, sad, fearful or angry / 1s control movies of dark grey oval morphing to larger shape; 12 stimuli/block; 3 blocks per emotion and 6 control blocks; 3000-3999ms inter-trial interval | Happy > control  Sad > control  Fearful > control  Angry > control |
| Walter et al. 2009 | Emotional stimulation | Positive / negative IAPS pictures; 4s picture viewing or picture judgement with or without 8-11.5s preceding expectancy period / 6-8s fixation cross; 159 trials | Negative + positive > fixation cross |

*IAPS = International Affective Picture System*

Table S5 Methodological details of studies of ACC glutamate and cognitive control-related neural activity

| Study | fMRI paradigm | Stimuli | Contrast |
| --- | --- | --- | --- |
| Brennan et al. 2015 | Emotional stroop | 1.5s set of 1-4 identical words (neutral / OCD-unrelated negative / OCD-specific negative) displayed simultaneously; participants indicated via button press the number of words displayed per trial; 20 trials/block; 3 blocks | OCD-specific negative > neutral words |
| Cadena et al. 2018 | Stroop | 1.5 s one of three words (“RED”, “GREEN”, “BLUE”) displayed in the same colour (congruent trial) or in a different colour (incongruent trial) where participants indicated the colour of the word / 1.5s fixation cross; 88 trials x 3 runs (~30% incongruent, ~70% congruent) | Incongruent > congruent |
| Falkenberg et al. 2012 | Bergen dichotic listening | A pair of consonant-vowel syllables with one syllable presented to each ear simultaneously; three voiced syllables (ba, da, ga) and three unvoiced syllables (ka, pa, ta), only syllables within the same voicing were paired; 400-500ms syllable presentation; a bottom-up component varied the salience with five levels of interaural intensity differences; a top-down component gave instructions to focus attention to the right or left ear stimulus (text on screen “Attend left/right ear” and an arrow) 1.5s before auditory stimuli; bottom-up and top-down component resulted in 10 experimental conditions with 18 presentations each; 180 stimulus presentations pseudorandomly intermixed with 90 silent null events; participants responded to dichotic presentation by orally naming the syllable they heard | GLM: attention instruction, interaural intensity difference, Glu |
| Overbeek et al. 2019 | Stroop | One of three words (“red”, “green”, “blue”) displayed in the same colour (congruent trial) or in a different colour (incongruent trial) where participants indicated the colour of the word; 64 trials x 3 runs (30% incongruent, 70% congruent) | Incongruent > congruent |
| Reid et al. 2010 | Stroop | 1.5 s one of three words (“RED”, “GREEN”, “BLUE”) displayed in the same colour (congruent trial) or in a different colour (incongruent trial) where participants indicated the colour of the word / 1.5s fixation cross; 88 trials | Incongruent > congruent |
| Yucel et al. 2007 | Multi-source interference | Set of numbers from 0 to 3 presented in strings of three with one number always being different to the other two; participants indicated numerical value of the number that was different; Congruent trials – the numerical value matched the position on the response button box and accompanied by two 0’s; Incongruent trials – the numerical value never matched the position on the response button box; 60s fixation cross at beginning and end of scan; 30s alternating condition blocks; 8 blocks | Incongruent > congruent |

*OCD = Obsessive-compulsive disorder*

Table S6 Methodological details of studies of ACC glutamate and emotion-related neural activity

| Study | fMRI paradigm | Stimuli | Contrast |
| --- | --- | --- | --- |
| Duncan et al. 2011 | Empathy | Happy / angry / disgusted / neutral expressions from Matsumoto and Ekman’s Japanese and Caucasian Facial Expressions of Emotion; smoothed pictures as control pictures; 5s pictures | Fixation > empathy  empathy > smoothed control |
| Levar et al. 2017b | Emotion processing | 20 negative / neutral / positive IAPS pictures; positive and negative matched for arousal; 4s stimuli / 3-5s fixation cross | Negative + positive > neutral |
| Northoff et al. 2007 | Emotion processing | Positive / negative IAPS pictures; 4s picture viewing or picture judgement with or without 8-11.5s preceding expectancy period / 6-8s fixation cross; 159 trials | Negative + positive > fixation cross |
| Modinos et al. 2017 | Emotional viewing | 10 IAPS pictures per category (negative high arousal, negative low arousal, positive high arousal, positive low arousal, neutral) matched for 50% social content; 4000ms picture / 1000-10 000ms fixation cross / 4000ms rating screen where participants indicate emotional arousal | Emotional > neutral |
| Stan et al. 2014 | Implicit emotion processing and regulation | 1s movies of facial expressions morphing from neutral to happy, sad, fearful or angry / 1s control movies of dark grey oval morphing to larger shape; 3 blocks per emotion and 6 control blocks | Happy > control  Sad > control  Fearful > control  Angry > control |
| Von During et al. 2019 | Salience processing with emotional and erotic pictures | 40 high salient erotic or low salient emotional IAPS pictures; 3-5s stimulus presentation / 7.5-10.5s fixation cross; two types of expectancy cues were presented: arrows to indicate content of following picture (upward – non-sexual emotional content; downward – erotic content) and dots to indicate the number of persons in the following picture | Expected emotional pictures > fixation  Unexpected emotional pictures > fixation |
| Walter et al. 2009 | Emotional stimulation | Positive / negative IAPS pictures; 4s picture viewing or picture judgement with or without 8-11.5s preceding expectancy period / 6-8s fixation cross; 159 trials | Negative + positive > fixation cross |

*IAPS = International Affective Picture System*

## Sensitivity analyses

### Occipital GABA and visual-related neural activity

Due to the various analyses on the same relationship, for example using %SC from the peak voxel or the entire ROI and using GABA+ as well as GABA- measures for the same analysis within a paper, a sensitivity analysis was conducted to gauge the range of the effect of this relationship (Fig. S1). Testing the maximum negative effect from each study, showed results in favour of a relationship between GABA and BOLD in the visual cortex (r=-0.47 [-0.61, -0.31], p < 0. 001, I^2^=9.45%). Similarly, testing the maximum positive effect from each study, the meta-analysis produced results in support of the negative relationship (r=-0.37 [-0.59, -0.12], p=0. 005, I^2^=56.18%).

Figure 1. Sensitivity analysis for occipital lobe ^1^H-MRS GABA and visual processing related neural activity.


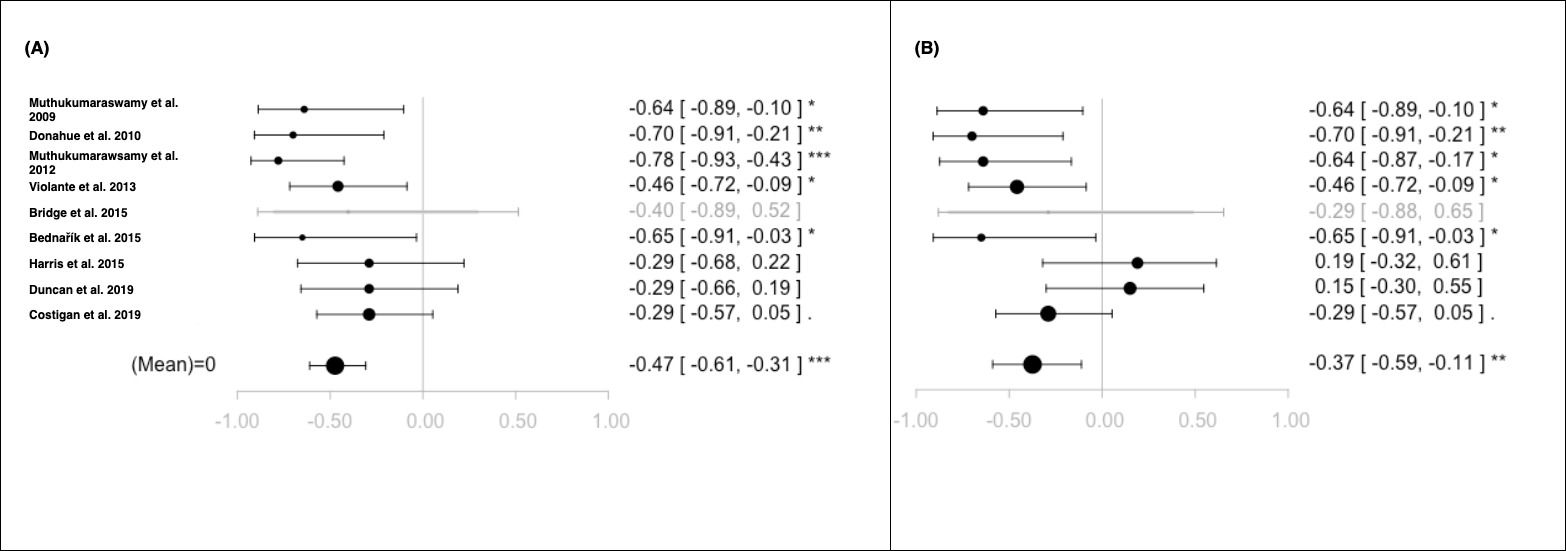


(A) Sensitivity analysis using maximum negative effects. (B) Sensitivity analysis using maximum positive effects. .*p* < 0.10; **p* < 0.05; ***p* < 0.01; ****p*<0.001

### ACC glutamate and cognitive control-related neural activity

For sensitivity analysis of studies examining ACC glutamate and cognitive control activity, neither the maximum nor the minimum effect sensitivity analyses yielded a significant relationship between the two measures (max: r=0.19 [-0.23, 0.55], p=0.39, I^2^=72.04%; min: r=0.01 [-0.23, 0.25], p=0.93, I^2^=2.89%) (Fig. S2).

Figure 2. Sensitivity analysis for ACC ^1^H-MRS glutamate and cognitive control-related neural activity


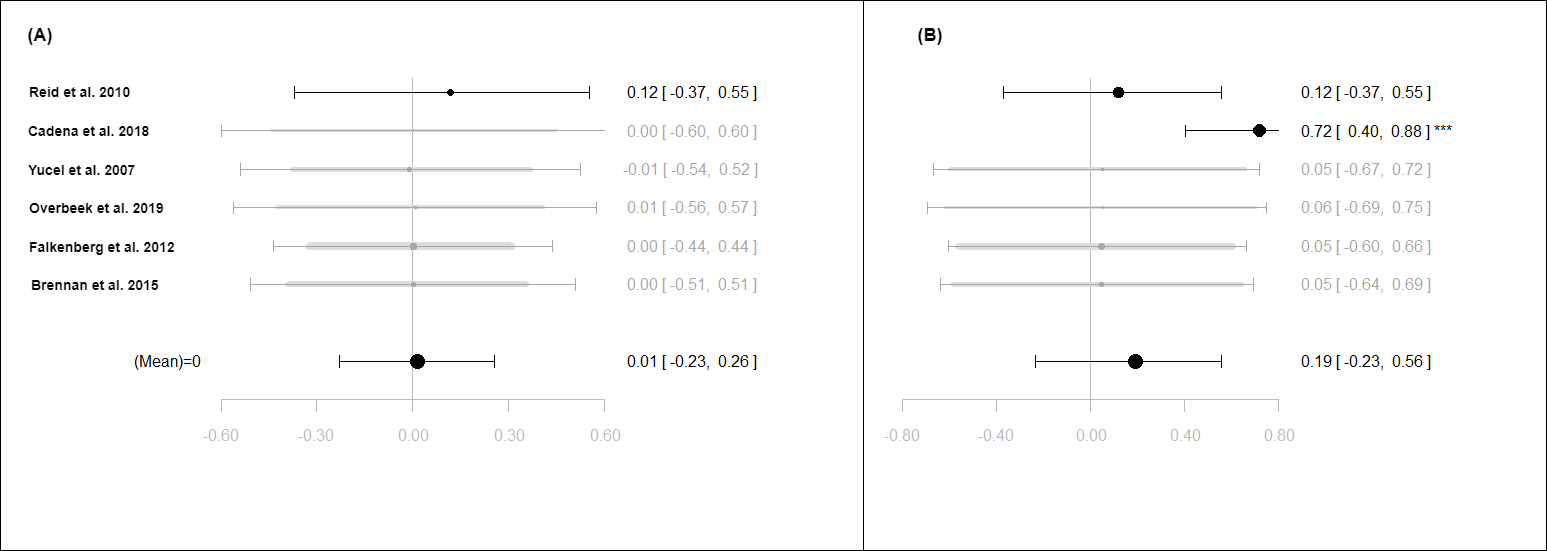


(A) Sensitivity analysis using maximum negative effects. (B) Sensitivity analysis using maximum positive effects. ****p*<0.001

### ACC glutamate and emotion processing-related neural activity

Both sensitivity analyses for ACC glutamate levels and emotion processing did not return significant effects. However, the maximum positive effect sensitivity analysis missed the significant threshold by a small amount (r=0.39 [-0.05, 0.72], p=0.08, I^2^=79.45%). Note that this is the same as the main analysis, as this analysis included the most homogenous analyses types. The most negative effect sensitivity analysis did not reach significance (r=0.05 [-0.18, 0.27], p=0.678, I^2^=7.19%) (Fig. S3).

Figure 3. Sensitivity analysis for ACC ^1^H-MRS glutamate and emotion processing-related neural activity.


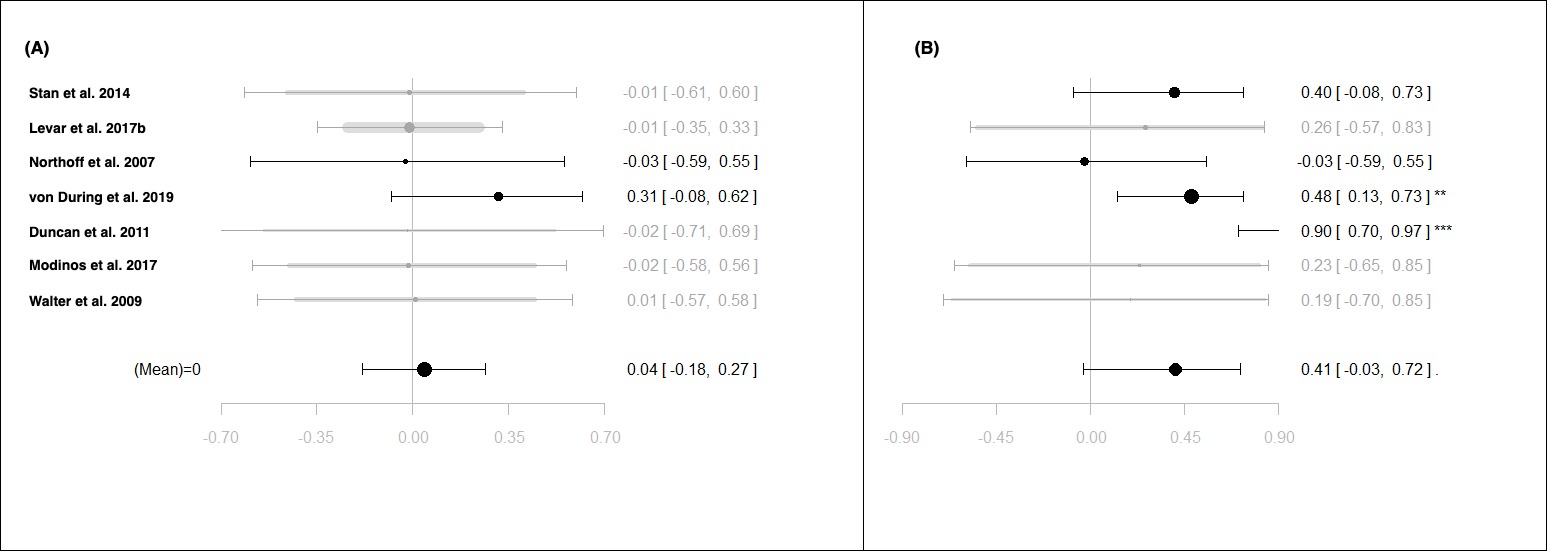


(A) Sensitivity analysis using most negative effects. (B) Sensitivity analysis using most positive effects. .*p* < 0.10; ***p* < 0.01; ****p*<0.001

## Control analyses

Table S7. GABA control analyses

| **Authors** | **sample sex (male) mean age (SD)** | **System**  **Sequence** | **Reference** | **MRS location** | **Combined with** | **Results Region OR ROI: Activation (Correlation) Metabolite** |
| --- | --- | --- | --- | --- | --- | --- |
| **Chen et al. 2019** | 19 H 6 m 24 (2) | 3T MEGA-PRESS | /Wt | mPFC | Working memory task (Sternberg item recognition paradigm) | dlPFC: any contrast (#) GABA |
| **Schallmo et al. 2018** | 27 H 12 m 24 (3.6) | 3T MEGA-PRESS | /Wt | Fronto-parietal cortex (control region) | Spatial suppression stimuli | Parietal lobe: suppression (#) GABA+ |
| **Schmitz et al. 2017** | 30 H (18) 7 m 24.7 (4.3) | 3T 2D-JPRESS | /Cr | dlPFC | Think/No-Think Task | dlPFC: Think (#) GABA dlPFC: No-Think (#) GABA |
| **Schmitz et al. 2017** | 30 H (18) 7 m 24.7 (4.3) | 3T 2D-JPRESS | /Cr | Hippocampus | Stop/Go Task (control task) | Hippocampus: Stop (#) GABA Hippocampus: Go (#) GABA |
| **Jung et al. 2017** | 20 H 7 m 23 (4) | 3T MEGA-PRESS | /NAA | L anterior temporal lobe | pattern matching task (control task) | L ATL: Pattern matching (#) GABA |
| *Wt = Water; Cr = Creatine; NAA = N-acetylaspartic acid; mPFC = medial prefrontal cortex; dlPFC = dorsolateral prefrontal cortex; L = left; ATL = anterior temporal lobe; GABA+ = GABA+macromolecules* | | | | | | |

| **Authors** | **sample sex (male) mean age (SD)** | **System Sequence** | **Reference** | **MRS location** | **Combined with** | **Results Region OR ROI: Activation (Correlation) Metabolite** |
| --- | --- | --- | --- | --- | --- | --- |
| **Costigan et al. 2019** | 40 H 12 m 22.1 (2.1) | 3T PRESS | /Wt | occipital lobe (control region) | odd-one-out task | R PCu/PCC: Scenes > Faces+Objects (#) Glx |
| **Stagg et al. 2011** | 12 H 6 m 23 (-) | 3T MEGA-PRESS | /NAA | visual cortex (control region) | Visually cued reaction time task | Whole brain: movement > rest (#) Glx |
| **Duncan et al. 2011** | 13 H 4 m 31.6 (-) | 3T PRESS | /Cr | perigenual anterior cingulate cortex | modified monetary incentive delay task (control task) | Subgenual ACC: reward anticipatioon (#) Glx |
| **Duncan et al. 2011** | 13 H 4 m 31.6 (-) | 3T PRESS | /Cr | perigenual anterior cingulate cortex | empathy evaluation task (control task) | Subgenual ACC: empathy evaluation (#) Glx |
| **Duncan et al. 2011** | 13 H 4 m 31.6 (-) | 3T PRESS | /Cr | L anterior Insula (control region) | empathy task | Cingulate cortex: empathy (#) Glx |
| **Duncan et al. 2011** | 13 H 4 m 31.6 (-) | 3T PRESS | /Cr | L anterior Insula (control region) | modified monetary incentive delay task (control task) | Cingulate cortex: reward anticipation (#) Glx |
| **Duncan et al. 2011** | 13 H 4 m 31.6 (-) | 3T PRESS | /Cr | L anterior Insula (control region) | empathy evaluation task (control task) | Cingulate cortex: empathy evaluation (#) Glx |
| **Duncan et al. 2015** | 12 H 6 m 23 (3.5) | 3T MEGA-PRESS | /NAA | L insula (control region) | Aversion Task | Whole: anticipation of certain aversion (#) Glu / Glx Whole: anticipation of uncertain aversion (#) Glu / Glx |
| **Enzi et al. 2012** | 19 H 10 m 29.6 (-) | 3T PRESS | /Cr | L anterior Insula (control region) | modified monetary incentive delay task | L aInsula: Any contrast (#) Glx |
| **Jung et al. 2017** | 20 H 7 m 23 (4) | 3T MEGA-PRESS | /NAA | L anterior temporal lobe | pattern matching task (control task) | L ATL: Pattern matching (#) Glx |
| **Northoff et al. 2007** | 12 H 4 m 33.8 (-) | 3T 2D-JPRESS | /Cr | R Paracentral cortex (control region) | Emotion processing task (negative BOLD responses) | Paracentral cortex: all contrasts (#) Glx |
| *Wt = Water; Cr = Creatine; NAA = N-acetylaspartic acid; L = left; R = right; PCu/PCC = precuneus/posterior cingulate cortex; ACC = anterior cingulate cortex; aInsula = anterior Insula; ATL = anterior temporal lobe; Glu = glutamate; Glx = glutamate + glutamine* | | | | | | |

**Table S8. Glutamate control analyses**

## Study parameters

Table S9. Study imaging and design parameters

| Study | Scanner Strength | MRS Sequence | Metabolite | Reference | Averages | TE (ms) | Voxel size | Data points | Shim or FWHM | Fit error (CRLB) | Data visualisation | Partial volume correction | Frequency or phase correction | Block design | Controlled for age? | Controlled for sex? | MRS control region? | Control task? | Analysis method |
| --- | --- | --- | --- | --- | --- | --- | --- | --- | --- | --- | --- | --- | --- | --- | --- | --- | --- | --- | --- |
| Allen et al. 2015 | 3 | PRESS | Glx | - | 96 | 30 | 15x20x20 | - | Y | 20 | N | GM, WM, CSF | - | Y | N | N | N | N | Regression |
| Bednařík et al. 2015 | 7 | semi-LASER | GABA, Glu | Wt | 768 | 26 | 20x20x20 | - | Y | 50 | Y | - | Y | Y | N | N | N | N | Correlation |
| Bhattacharyya et al. 2013 | 3 | MEGA-PRESS | GABA | Wt | 96 | 68 | 20x20x20 | - | - | - | Y | GM, WM, CSF | Y | Y | N | N | N | N | ROI regression |
| Bhattacharyya et al. 2017 | 3 | MEGA-PRESS | GABA | Wt | 96 | 68 | 20x20x20 | - | - | - | Y | GM, WM, CSF | Y | Y | N | N | N | N | ROI regression |
| Bossong et al. 2018 | 3 | PRESS | Glx | Wt | 96 | 30 | 20x20x15 | - | Y | 20 | N | GM, WM, CSF | - | N | N | N | N | N | Correlation |
| Brennan et al. 2015 | 3 | 2D-JPRESS | Glu | tCr | 16 | 35-350 | 20x20x20 | 64 | Y | - | Y | Given, but not corrected | - | N | Y | Y | N | N | Correlation |
| Bridge et al. 2015 | 3 | SPECIAL | GABA, Glu | tCr | 128 | 85 | 20x20x20 | - | Y | 20 | N | GM, WM, CSF | Y | Y | N | N | N | N |  |
| Cadena et al. 2018 | 3 | PRESS | Glx | Cr | 256 | 80 | 27x20x10 | - | Y | 20 | N | - | - | N | N | N | N | N | ROI regression |
| Chen et al. 2019 | 3 | MEGA-PRESS | GABA | Wt | 192 | 68 | mPFC: 30x20x30 dlPFC: 25x25x25 | - | Y | - | Y | GM, WM, CSF | Y | N | N | Y | Y | N | Multiple regressor model |
|  |  | Semi-LASER | Glu | Wt | 64 | 28 | mPFC: 30x20x30 dlPFC: 25x25x25 | - | Y | - | Y | GM, WM, CSF | Y | N | N | Y | Y | N | Multiple regressor model |
| Cleve et al. 2017 | 3 | MEGA-PRESS | GABA, Glx | tCr | 384 | 68 | 28x40x14 | - | Y | - | Y | GM, WM | - | Y | N | N | N | N |  |
| Costigan et al. 2019 | 3 | MEGA-PRESS | GABA+ | Wt | PCC: 256 Occ: 166 | 68 | PCC: 20x20x30 Occ: 30x30x30 | - | Y | 20 | Y | GM, WM, CSF | - | Y | N | N | Y | N | Correlation & ROI Regression |
|  |  | PRESS | Glx | Wt | 128 | 35 | PCC: 20x20x30 Occ: 30x30x30 | - | Y | 20 | Y | GM, WM, CSF | - | Y | N | N | N | N | Correlation & ROI Regression |
| Donahue et al. 2010 | 3 | MEGA-PRESS | GABA+ | Cr | 192 | 69 | 30x30x30 | - | Y | - | Y | - | Y | Y | N | N | N | N | Correlation & ROI Regression |
| Draper et al. 2014 | 7 | STEAM | GABA | Wt | 288 | 16 | 20mm^3^ | 4096 | Y | 30 | N | GM | Y | Y | N | N | N | N | Correlation & ROI Regression |
| Duncan et al. 2019 | 3 | MEGA-PRESS | GABA+, GABA- | Wt | 192 | GABA+: 68 GABA-: 80 | 30x30x30 | 4096 | Y | Y | Y | GM, WM, CSF | Y | Y | N | N | N | N | Regression |
| Duncan et al. 2011 | 3 | PRESS | Glx | Cr | - | 80 | pgACC&sgACC: 20x10x20 Insula: 15x10x20 | - | Y | 20 | N | - | - | N | N | N | Y | Y | Regression |
| Duncan et al. 2015 | 3 | MEGA-PRESS | Glx | NAA | - | - | mPFC: 48x21x21 Insula: 23x48x27 | - | - | 20 | N | - | - | N | N | N | Y | N | Correlation & ROI Regression |
| Enzi et al. 2012 | 3 | PRESS | Glx | Cr | - | 80 | pACC: 20x10x20 Insula: 15x10x20 | - | - | 20 | N | - | - | N | Y | N | Y | N | Correlation |
| Falkenberg et al. 2012 | 3 | PRESS | Glu | Cr | 128 | 35 | 24x20x20 | - | Y | - | N | Tested correlation to GM and WM, but not corrected | - | N | Y* | N | N | N | Regression |
| Friedman et al. 2017 | 3 | MEGA-PRESS | GABA | tCr | 128 | 68 | 30x30x30 | - | - | Y | Y | GM/WM reported | - | Y | N | N | N | N | ROI regression |
| Fusar-Poli et al. 2011 | 3 | PRESS | Glu | - | 96 | 30 | ACC: 20x20x20 Hippocampus: 20x20x15 Thalamus: 15x20x20 | - | Y | 20 | N | GM, WM, CSF | - | Y | N | N | N | N | Regression |
| Gleich et al. 2015 | 3 | PRESS | Glu | - | 128 | 80 | 20x30x25 | - | Y | Y | Y | GM, WM, CSF | - | N | N | N | N | N | Correlation |
| Harris et al. 2015 | 3 | MEGA-PRESS | GABA+ | Cr, Wt | 320 | 68 | 30x30x30 Auditory cortex: 40x30x20 | 2048 | - | - | Y | GM, WM | Y | Y | N | N | N | N | Correlation |
| Hu et al. 2013 | 3 | MEGA-PRESS | GABA+ | Wt | 96 | 68 | 24x32x36 | - | Y | 20 | Y | GM, WM, CSF | - | Y | Y | N | N | N | Multiple regressor model |
|  | 3 | PRESS | Glu | Wt | 128 | 30 | 24x32x36 | - | Y | 20 | Y | GM, WM, CSF | - | Y | Y | N | N | N | Multiple regressor model |
| Hutcheson et al. 2012 | 3 | PRESS | Glx | Cr | 640 | 80 | 27x15x10 | - | Y | 30 | Y | - | - | N | N | N | N | N | Correlation |
| Ip et al. 2017 | 7 | semi-LASER | Glu | tCr | 728 | 36 | 20x20x20 | - | - | 20 | Y | CSF | Y, | Y | N | N | N | N |  |
| Jocham et al. 2012 | 3 | SPECIAL | GABA | Cr | vmPFC: 180 Parietal: 360 | 8.5 | vmPFC: 15x30x10 Parietal: 20x20x20 | - | - | - | Y | GM | Y | N | N | N | N | N | Regression |
| Jung et al. 2017 | 3 | MEGA-PRESS | GABA, Glx | NAA | ATL: 79 Occ: 74 | 68 | ATL: 35x25x15 Occ: 30x30x30 | 1042 | - | - | Y | Reported and tested in relation to BOLD | - | Y | N | N | N | Y | Correlation & ROI regression |
| Kaminski et al. 2019 | 3 | PRESS | Glu | Wt | 128 | 80 | 40x10x20 | - | Y | 20 | Y | GM, WM | - | Y | Y | N | N | N | Correlation |
| Levar et al. 2017a | 3 | MEGA-PRESS | GABA+ | Cr | 384 | 73 | 40x20x20 | - | Y | - | Y | - | Y | N | N | N | N | N | Regression |
| Levar et al. 2017b | 3 | MEGA-PRESS | GABA+ | Cr | 384 | 73 | 40x20x20 | 1042 | Y | - | N | - | - | N | N | N | N | N | Regression |
| Lipp et al. 2015 | 3 | MEGA-PRESS | GABA+ | Wt | 300 | 68 | 25x30x40 | 4096 | - | - | N | GM, WM, CSF | - | Y | N | N | N | N | Regression & Correlation |
| Lorenz et al. 2015 | 3 | PRESS | Glx | Wt | 128 | 80 | 20x20x20 | - | Y | 30 | N | GM, WM, CSF | - | N | N | N | N | N | Correlation |
| Modinos et al. 2017 | 3 | PRESS | Glu | Wt | 96 | 30 | 20x20x20 | - | Y | 20 | Y | GM, WM, CSF | - | N | N | N | N | N | ROI Regression |
| Moon & Joeng2018 | 3 | PRESS | Glx | Cr | 969 | 30 | 20x20x20 | 1024 | - | 30 | Y | - | - | N | N | N | N | N | Correlation |
| Muthukumaraswamy et al. 2009 | 3 | MEGA-PRESS | GABA | Wt | 512 | 68 | 30x30x30 | - | - | - | Y | - | - | Y | N | N | N | N | Correlation |
| Muthukumaraswamy et al. 2012 | 3 | MEGA-PRESS | GABA | Wt | 512 | 68 | 30x30x30 | 2000 | - | - | Y | - | - | Y | N | N | N | N | Correlation |
| Naaijen et al. 2018 | 3 | PRESS | Glu | Cr | 96 | 30 | 20x20x20 | 4096 | - | 20 | Y | Not corrected, but checked for consistency | - | N | N | N | N | N | Correlation |
| Northoff et al. 2007 | 3 | 2D-JPRESS | GABA | Cr | 400 | 31-229 | ACC: 20x25x35 Paracentral cortex: 25x25x25 | 2048 | - | 20 | Y | - | - | N | N | N | Y | N | Correlation |
| Overbeek et al. 2019 | 7 | STEAM | Glu, GABA | Wt | 32 | 5 | 27x20x10 | 2048 | Y | Y | Y | GM, WM, CSF | - | N | N | N | N | N | Regression |
| Reid et al. 2010 | 3 | PRESS | Glx | Cr | 256 | 80 | 27x20x10 | - | Y | 30 | Y | - | - | N | N | N | N | N | Correlation |
| Schallmo et al. 2018 | 3 | MEGA-PRESS | GABA+ | Wt | 320 | 68 | 30x30x30 | 2048 | - | - | N | GM, WM | Y | Y | N | N | Y | N | Correlation |
| Schallmo et al. 2019 | 3 | MEGA-PRESS | Glx | Wt | 320 | 68 | 30x30x30 | 2048 | - | - | N | GM, WM | Y | Y | N | N | N | N | Correlation |
| Schmitz et al. 2017 | 3 | 2D-JPRESS | GABA, Glu | Cr | 400 | 31-229 | - | 1024 | Y | - | Y | Calculated, but not corrected | - | N | N | Y | Y | Y | Correlation |
| Stagg et al. 2011 | 3 | MEGA-PRESS | GABA, Glx | NAA | 256 | 68 | Sensorimotor: 20x20x20 Occ: 20x30x20 | - | - | - | Y | GM, WM, CSF | - | Y | N | N | Y | N | Regression & Correlation |
| Stan et al. 2014 | 3 | PRESS | Glu | Cr | 64 | 30 | 15x15x15 | - | Y | Y | Y | - | - | N | Y | N | N | N |  |
|  | 3 | MEGA-PRESS | GABA+ | Cr | 256 | 68 | 40x40x15 | - | Y | - | Y | - | - | N | N | N | N | N | ROI Regression |
| Valli et al. 2011 | 3 | PRESS | Glu | Wt | 96 | 30 | ACC: 20x20x20 Hippocampus: 20x20x15 Thalamus: 15x20x20 | - | Y | 20 | Y | GM, WM, CSF | - | Y | N | N | N | N | Correlation |
| Violante et al. 2013 | 3 | MEGA-PRESS | GABA, Glx | tCr | 196 | 68 | 3cm^3^ | 1024 | - | - | Y | - | - | N | Y* | Y* | N | N | Correlation |
| von During et al. 2019 | 7 | STEAM | Glu | tCr | 128 | 20 | 25x15x10 | - | Y | 20 | N | - | - | N | Y | Y | N | N | Regression & Correlation |
| Walter et al. 2009 | 3 | 2D-JPRESS | GABA, Glu | Cr | 400 | 31-229 | 20x25x35 | 2048 | - | 20 | N | - | - | N | Y | Y | N | N | Correlation |
| Wang et al. 2017 | 3 | MEGA-PRESS | GABA | - | 96 | 68 | 40x30x20 | 2048 | - | - | Y | GM, WM, CSF | - | N | Y* | N | N | N | ROI Regression |
| White et al. 2015 | 3 | PRESS | Glx | Cr | 640 | 80 | 13x13x13 | 1024 | Y | 30 | Y | - | - | N | N | N | N | N | ROI Regression |
| Wiebking et al. 2014 | 3 | MEGA-PRESS | GABA, Glx | NAA | - | - | mPFC: 48x21x21 Insula: 23x48x27 | - | Y | 20 | Y | - | Y | N | N | N | N | N | Correlation & ROI regression |
| Wijtenburg et al. 2017 | 3 | STEAM | Glu | Wt | 128 | 6.5 | 30x40x20 | 2048 | Y | 20 | Y | GM, WM, CSF | Y | Y | Y | N | N | N | ROI Regression & Correlation |
|  | 3 | MEGA-PRESS | GABA- | Wt | 256 | 68 | 30x40x20 | 1024 | Y | - | Y | GM, WM, CSF | Y | Y | Y | N | N | N |  |
| Witt et al. 2018 | 3 | MEGA-PRESS | GABA+, Glu | Wt | - | 68 | 30x30x30 | - | - | - | N | - | Y | N | N | N | N | N | Correlation |
| Yucel et al. 2007 | 3 | PRESS | Glx | - | 128 | 30 | 6.5cm^3^ | - | Y | Y | N | - | - | Y | N | N | N | N | Correlation |
| *Fit error (CRLB): Y = Fit error reported without threshold value given; Controlled for age / sex?: Y* = GABA or BOLD tested for correlation with age / sex; GM = grey matter volume fraction; WM = white matter volume fraction; CSF = cerebrospinal fluid volume fraction; Glu = glutamate; Glx = glutamate + glutamine; GABA+ = GABA+macromolecules; Wt = water; Cr = createine; tCr = creatine+phosphocreatine; NAA = N-acetylaspartic acid; mPFC = medial prefrontal cortex; dlPFC = dorsolateral prefrontal cortex; vmPFC = ventromedial prefrontal cortex; Occ = occipital lobe; PCC = posterior cingulate cortex; sgACC = supragenual ACC; pgACC = perigenual ACC; pACC = posterior ACC; ATL = anterior temporal lobe* | | | | | | | | | | | | | | | | | | | |
